# Identification of a flavonoid *C*-glycosyltransferase from fern species *Stenoloma chusanum* and the application in synthesizing flavonoid *C*-glycosides in *Escherichia coli*

**DOI:** 10.1186/s12934-022-01940-z

**Published:** 2022-10-14

**Authors:** Rong Ni, Xin-Yan Liu, Jiao-Zhen Zhang, Jie Fu, Hui Tan, Ting-Ting Zhu, Jing Zhang, Hai-Long Wang, Hong-Xiang Lou, Ai-Xia Cheng

**Affiliations:** 1 Key Laboratory of Chemical Biology of Natural Products, Ministry of Education, School of Pharmaceutical Sciences, Shandong University, Jinan, China; 2 State Key Laboratory of Microbial Technology, Institute of Microbial Technology, Helmholtz International Lab for Anti-Infectives, Helmholtz Institute of Biotechnology, Shandong University, Qingdao, China

**Keywords:** *C*-glycosides, *Stenoloma chusanum*, *C*-glycosyltransferases, Orientin, Vitexin, Metabolic engineering

## Abstract

**Background:**

Flavonoid *C*-glycosides have many beneficial effects and are widely used in food and medicine. However, plants contain a limited number of flavonoid *C*-glycosides, and it is challenging to create these substances chemically.

**Results:**

To screen more robust *C*-glycosyltransferases (CGTs) for the biosynthesis of flavonoid *C*-glycosides, one CGT enzyme from *Stenoloma chusanum* (ScCGT1) was characterized. Biochemical analyses revealed that ScCGT1 showed the *C*-glycosylation activity for phloretin, 2-hydroxynaringenin, and 2-hydroxyeriodictyol. Structure modeling and mutagenesis experiments indicated that the glycosylation of ScCGT1 may be initiated by the synergistic action of conserved residue His26 and Asp14. The P164T mutation increased *C*-glycosylation activity by forming a hydrogen bond with the sugar donor. Furthermore, when using phloretin as a substrate, the extracellular nothofagin production obtained from the *Escherichia coli* strain ScCGT1-P164T reached 38 mg/L, which was 2.3-fold higher than that of the wild-type strain. Finally, it is proved that the coupling catalysis of CjFNS I/F2H and ScCGT1-P164T could convert naringenin into vitexin and isovitexin.

**Conclusion:**

This is the first time that *C-*glycosyltransferase has been characterized from fern species and provides a candidate gene and strategy for the efficient production of bioactive *C*-glycosides using enzyme catalysis and metabolic engineering.

**Supplementary Information:**

The online version contains supplementary material available at 10.1186/s12934-022-01940-z.

## Background

Flavonoids are important phytochemicals that are widely distributed in plants and protect plants from various biotic and abiotic stresses [1, 2]. In plants, glycosylation of flavonoids is considered to play key roles in changing their physiological properties, including solubility and bioavailability [3, 4]. The sugar moieties can be linked to the skeleton of flavonoids through C–C, C–O, C–S, or C–N bonds to form *C*-glycosides, *O*-glycosides, *S*-glycosides, and *N*-glycosides, respectively [5]. Compared with *O*-glycosylated flavonoids, *C*-glycosides are more stable against acid hydrolysis and glycosidase [6, 7]. Furthermore, they exhibited various pharmacological properties, including antidiabetic, antioxidant, and antihypertensive activities [8, 9]. *C*-glycosyltransferases (CGTs), could combine an acceptor molecule, such as a 2-hydroxyflavanone or a flavone, with an activated sugar moiety, such as UDP-sugar, to create a variety of *C*-glycosylated natural products, which are members of the family of UDP-dependent glycosyltransferases (UGTs). Based on their substrate structures, plant CGTs could be categorized into 2 types: type I and type II (Additional file 1: Fig. S1) [10]. Type I CGTs attach one glucosyl moiety to a 2-hydroxyflavanone aglycone, which is formed by the hydroxylation at the C2 of the C-ring under the catalysis of flavanone 2-hydroxylase (F2H) [7]. After conjugation of the sugar moiety, the products (2-hydroxylflavanone *C*-glycoside) will produce flavone *C*-glycosides by enzymatic reaction or spontaneous dehydration. Most CGTs from this group act upon an open-ring form of 2-hydroxyflavanones [11]. In contrast, only ZmUGT708A6 from *Zea mays* can catalyze the *C*-glycosylation of closed-circular 2-hydroxynaringenin [12]. Type II CGTs directly transfer a sugar moiety to C-6 or C-8 of a flavone or isoflavone aglycone. So far, such enzymes have only been reported in *Gentiana trifloral* [13], *Eutrema japonicum* [14], *Trollius chinensis* [15], and *Pueraria lobata* [16].

In the last decade, research on plant CGTs has received a great deal of attention and achieved significant strides in the biosynthesis of natural and unnatural *C*-glycosides. To meet the synthetic requirements, the co-expression of cytochrome P450 F2H and CGT were applied for the production of flavonoid *C*-glycosides in *Escherichia coli* or *Saccharomyces cerevisiae* [17, 18]. For example, a flavone *C*-glycoside pathway was reconstructed in yeast by introducing P450 F2H and CGT genes from *Oryza sativa* [17]. In addition, Chen *et al*. [19] proposed the de novo biosynthesis of flavone di-*C*-glycosides with arabinosyl or other pentosyl groups in *E. coli*. To further improve the titer of flavone *C*-glycosides, one-step whole-cell synthesis in *E. coli* was employed [20, 21]. Many researchers prefer to use GtUF6CGT1 or TcCGT1, which were functionally characterized directly to conjugate glucose unit at the C-6 or C-8 of apigenin and luteolin, to produce vitexin, isovitexin, orientin, and isoorientin in vivo [20, 22]. However, there are far from meeting the requirement for producing in practical quantities of industrial production, so more CGT genes need to be excavated.

Flavonoid *C*-glycosides are widely existed in plants [23, 24], and around 70 CGTs have been characterized in monocotyledons [16, 25] and dicotyledons [26, 27]. Recently, GgCGT, a highly efficient di-*C*-glycosyltransferase, was discovered in *Glycyrrhiza glabra* [28]*.* Through structural analysis and molecular docking, Ye *et al.* found that the sugar donor selectivity and catalytic efficiency of GgCGT were affected by the hydrogen-bond interactions between sugar hydroxyl groups and Asp390. Although CGTs have been known and studied for years, more CGTs are required to synthesize *C*-glycosides. *Stenoloma chusanum* is a traditional Chinese medicinal herb, which belongs to the family of Lindsaeaceae. Two bioactive *C*-glycosides orientin and vitexin have been isolated from *S. chusanum* [29]. However, the corresponding CGTs have not been studied. In this study, one 2-hydroxyflavanone CGT was identified from *S. chusanum*. Molecular docking and site-directed mutagenesis were conducted to further study the active sites affecting ScCGT1 *C*-glycosylation. In addition, we took advantage of our previous research to co-express a soluble bifunctional CjFNS I/F2H protein with ScCGT1, which offered a useful system to synthesize flavone *C*-glycosides in *E. coli*.

## Results

### Analysis of flavonoids of *S. chusanum*

**Fig. 1:**
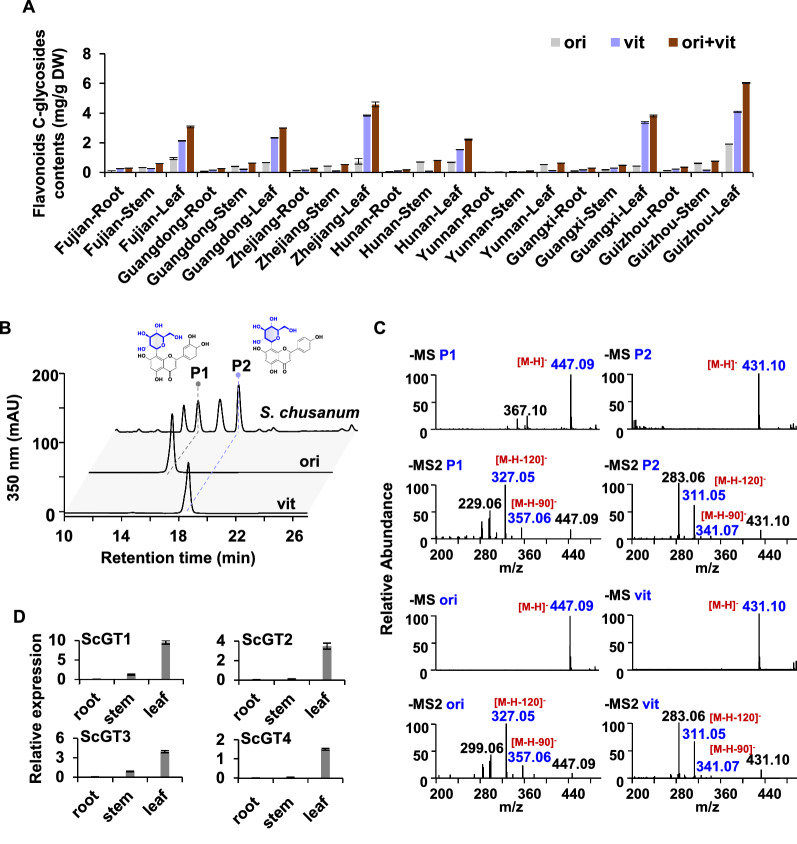
Analysis of flavonoids *C*-glycosides contents and gene expression in *Stenoloma chusanum*. **A** Relative levels of flavonoids *C*-glycosides [vitexin (vit) and orientin (ori)] in *S. chusanum.* **B** Representative HPLC chromatograms of methanolic extracts of *S. chusanum*. **C** Mass spectrometric profiles of P1, P2, and standards. **D** Expression analysis of candidate genes in *S. chusanum* organs. (P1: orientin; P2: vitexin; MS: mass spectrometry; MS2: tandem mass spectrometry)

Flavonoid profiles of *S. chusanum* from seven representative provinces were analyzed (Fig. 1A). As shown in Fig. 1B, C, compounds P1 and P2 shared the same retention time and mass spectrometry with the standards of orientin and vitexin, respectively. By comparing the content of *C*-glycosides of *S. chusanum* collected from different provinces, it was found that vitexin and orientin were the most abundant in the *S. chusanum* from Guizhou. Meanwhile, the corresponding compounds were mainly accumulated in leaves and less distributed in stems and roots (Fig. 1A). Additionally, the distribution of vitexin and orientin was compatible with the results of the expression levels of these four candidate genes in different tissues, which showed that they were predominantly concentrated in leaves (Fig. 1D).

### Biochemical characterization of ScGTs

The recombinant proteins of ScGT1, 2, 3, and 4 (Genbank accession number: ON936072, ON936073, ON936074, and ON936075; Additional file 2: Table S1) all contained fused Trx·tag, 6 × His·tag, S·tag, and the molecular weight was thus assigned as 72.67, 73.23, 72.80, and 75.31 kDa, respectively. The expressed recombinant proteins were purified with a Ni-affinity column and analyzed with SDS-PAGE (Additional file 1: Fig. S2).

**Fig. 2:**
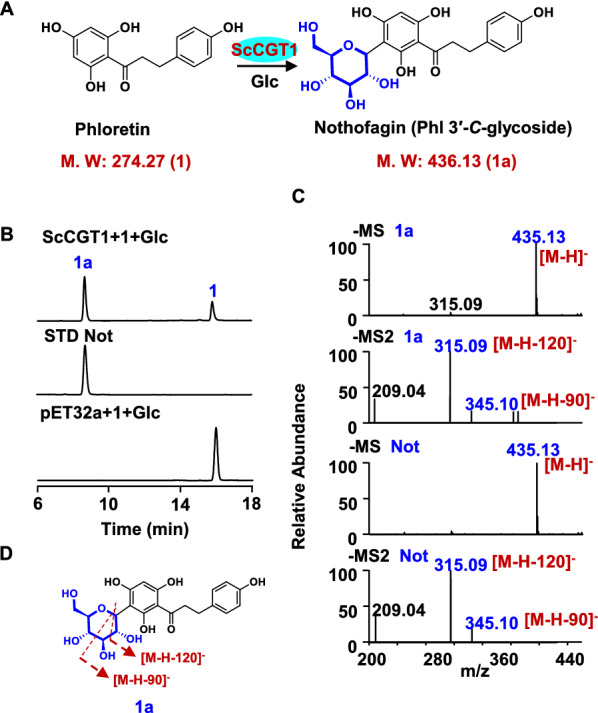
*C*-glycosylation of phloretin (1) catalyzed by recombinant ScCGT1. **A** ScCGT1 catalyzed phloretin (1) to produce 1a. **B** HPLC chromatograms of the glycosylation reactions. **C** Typical negative ion MS and MS2 spectra of product 1a. **D** The putative splitting way of 1a on MS2. (1, phloretin; Glc, UDP-glucose; Not: nothofagin; STD: Standard; MS: mass spectrometry; MS2: tandem mass spectrometry)

The catalytic activities of ScGTs were tested towards the substrates of apigenin, luteolin, phloretin, 2-hydroxynaringenin, and 2-hydroxyeriodictyol. Among them, only ScGT1 exhibited glycosylation activity against phloretin (1), 2-hydroxynaringenin (2), and 2-hydroxyeriodictyol (3). When phloretin was used as the substrate, a new peak (1a) was produced (Fig. 2). Mass spectrometry analysis of 1a showed that the molecular ion was m/z 435 [M − H] ^−^, which was 162 amu more than that of phloretin, indicating that this product was a mono-glycoside. According to the previous reports [30], the characteristic MS/MS fragment ions of flavonoid *C-*glycosides are [M‒H‒120]^−^ and [M‒H‒90]^−^. Hence, the fragment ions at m/z 345 [M‒H‒90]^−^ and m/z 315 [M‒H‒120]^−^ illustrated that this product was a *C*-glycoside (Fig. 2). Additionally, the retention time and mass spectrum of 1a was consistent with nothofagin (Fig. 2B, C). These results suggested that ScGT1 functioned as a *C*-glycosyltransferase. Thus, ScGT1 was then renamed ScCGT1.

**Fig. 3:**
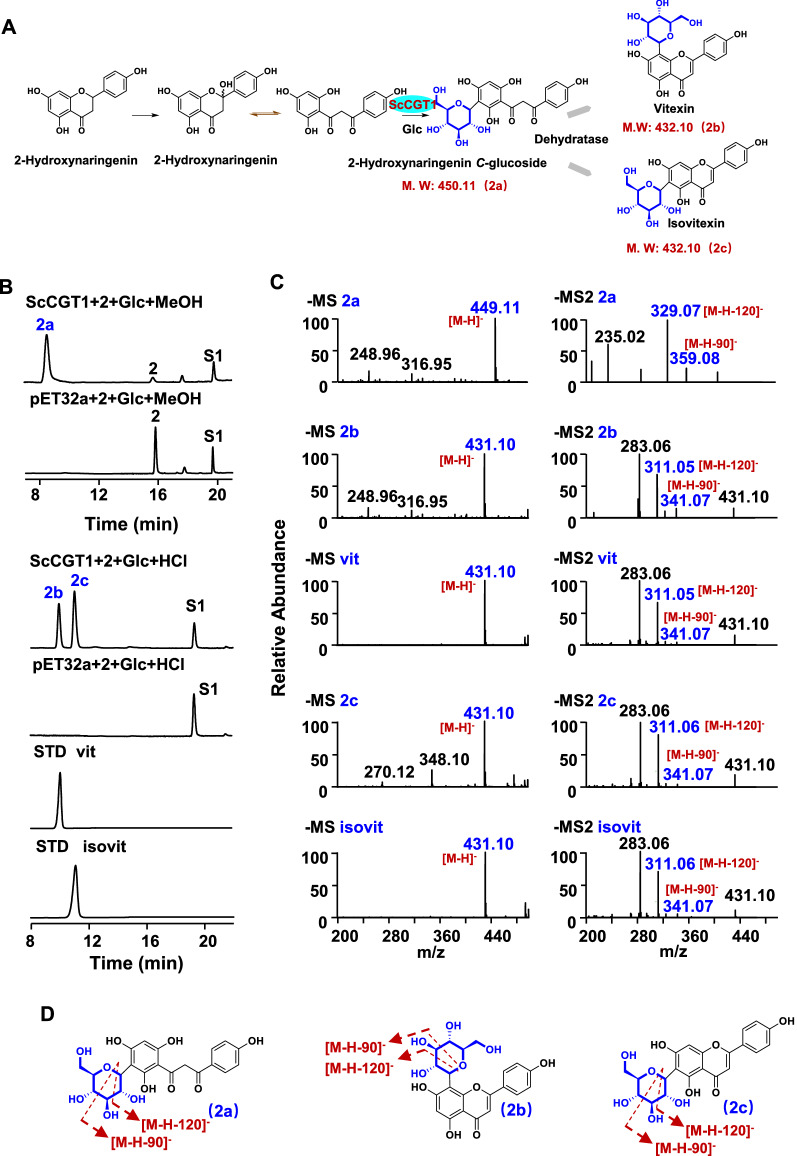
*C*-glycosylation of 2-hydroxynaringenin (2) catalyzed by recombinant ScCGT1. **A** ScCGT1 catalyzed 2-hydroxynaringenin (2) to produce 2a, 2b, and 2c. **B** HPLC chromatograms of the glycosylation reactions. **C** Typical negative ion MS and MS2 spectra of product 2a, 2b, 2c and standards. D. The putative splitting way of 2a, 2b, and 2c on MS2. S1, apigenin (dehydrated substrate); 2a, 2-hydroxynaringenin *C*-glycoside; 2b, vitexin; 2c, isovitexin. (2, 2-hydroxynaringenin; Glc, UDP-glucose; vit, vitexin; isovit, isovitexin; STD: Standard; MS: mass spectrometry; MS2: tandem mass spectrometry)

**Fig. 4:**
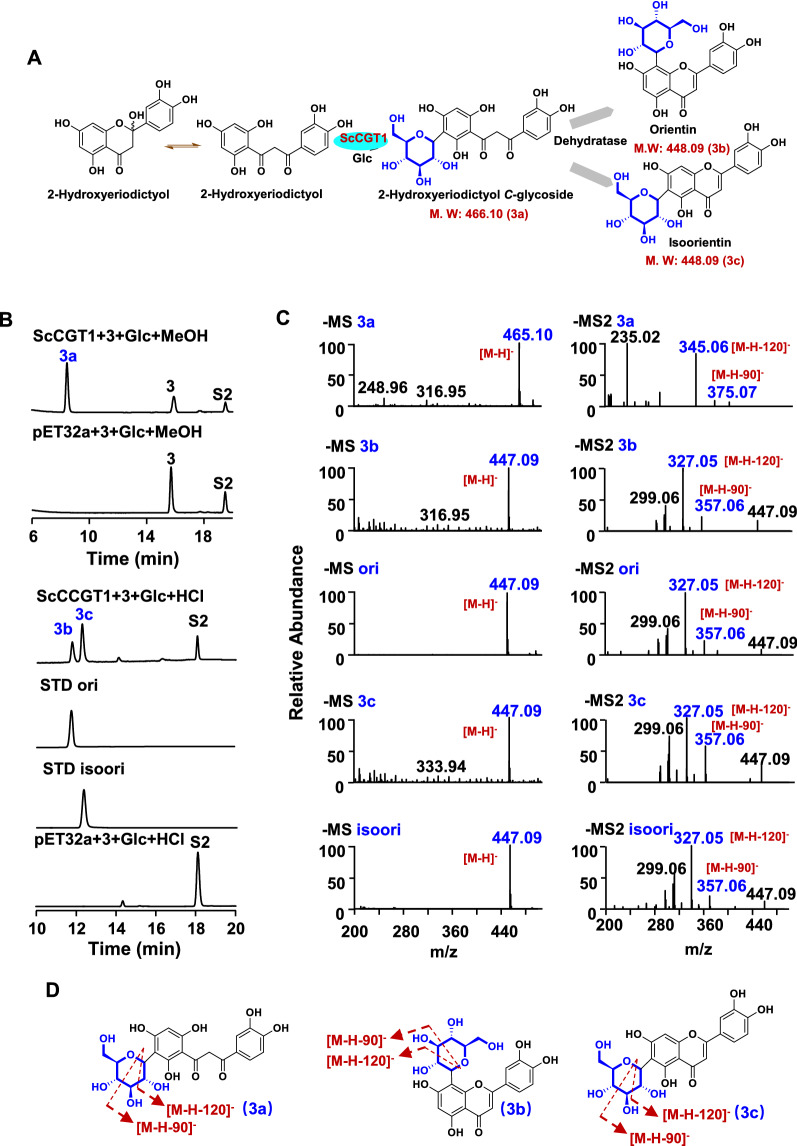
*C*-glycosylation of 2-hydroxyeriodictyol (3) catalyzed by recombinant ScCGT1. **A** ScCGT1 catalyzed 2-hydroxyeriodictyol (3) to produce 3a, 3b, and 3c. **B** HPLC chromatograms of the glycosylation reactions. **C** Typical negative ion MS and MS2 spectra of products 3a, 3b, 3c and standards. **D** The putative splitting way of 3a, 3b, and 3c on MS2. S2, luteolin (dehydrated substrate); 3a, 2-hydroxyeriodictyol *C*-glycoside; 3b, orientin; 3c, isoorientin. (3, 2-hydroxyeriodictyol; Glc, UDP-glucose; ori, orientin; isoori, isoorientin; STD: Standard; MS: mass spectrometry; MS2: tandem mass spectrometry)

When 2-hydroxynaringenin was used as the substrate, a major peak 2a was obtained (Fig. 3). The fragment ions, [M‒H]^−^ at m/z 449, [M‒H‒90]^−^ at m/z 359, and [M‒H‒120]^−^ at m/z 329, of 2a, suggested that this product was 2-hydroxynaringenin *C*-glycoside (Fig. 3B, C). After HCl was added to terminate the reaction, the dehydrated derivatives (2b and 2c) of 2a were detected in the established HPLC chromatography (Fig. 3). The retention time and mass spectrum of 2b and 2c were consistent with vitexin and isovitexin, respectively (Fig. 3B, C). Similarly, ScCGT1 also produced the 2-hydroxyeriodictyol *C*-glycoside (3a) following reaction with 2-hydroxyeriodictyol (Fig. 4). After treatment with HCl, two peaks (3b and 3c) were generated, which showed the same retention time and mass spectrum with orientin and isoorientin, respectively (Fig. 4). In contrast, ScGT2, 3, and 4 showed no activities with any substrates tested in this study.

To investigate the sugar donor selectivity, two additional UDP-sugar donors (UDP-galactose and UDP-glucuronic acid) were tested. ScCGT1 showed a weak activity toward UDP-galactose, which was approximately 10-fold lower than for UDP-glucose. However, when UDP-glucuronic acid was used as the donor, no product was detected (Additional file 1: Fig. S3).

### Phylogenetic and multiple sequence alignment analysis of ScCGT1

**Fig. 5:**
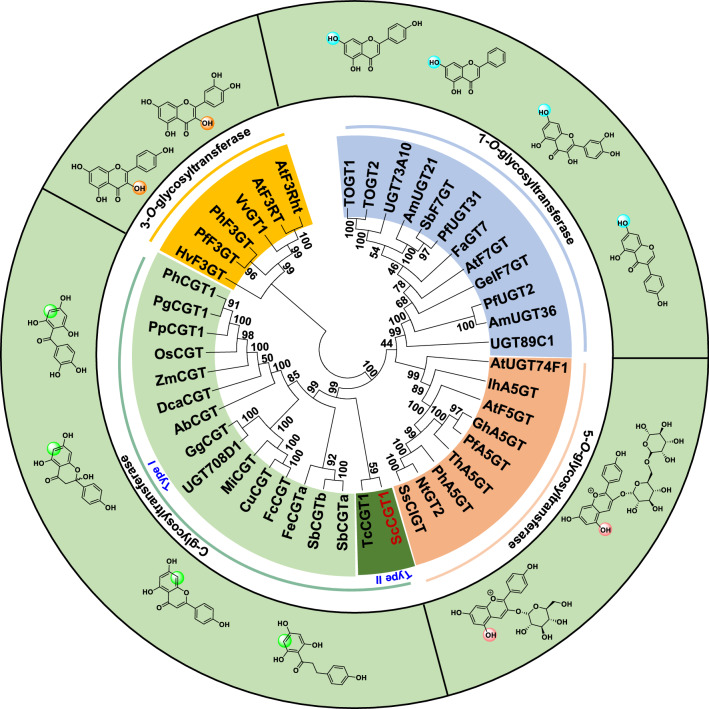
Phylogenetic analyses of the UDP-dependent glycosyltransferase family in plants. The tree was constructed using the ClustalW algorithm, based on the neighbor-joining algorithm with the aid of 1000 bootstrap replicates. The length of each branch reflects the distance between nodes. The abbreviations for species and accession numbers are listed in Additional file 2: Table S2

The phylogenetic analysis indicated that the UGTs were grouped into four major branches, including flavonoid 3-*O*-glycosyltransferases (3GTs), flavonoid 5-*O*-glycosyltransferases (5GTs), flavonoid 7-*O*-glycosyltransferases (7GTs), and flavonoid *C*-glucosyltransferases (CGTs). The results showed that ScCGT1 was grouped into the branches of CGTs (Fig. 5). Intriguingly, although ScCGT1 and type I CGTs, like GgCGT, both belonged to an orthologous group of enzymes that catalyze the *C*-glycosylation using the signature substrates as glycosyl acceptors, such as closed- or open-circular 2-hydroxyflavanones, or 2,4,6-trihydroxybenzophenone-like core structures, they were not closely grouped. It was partly because ScCGT1 shared low sequence similarity (18–28% amino acid identity) with others (Additional file 1: Fig. S4), and was thus distinct from type I CGTs in the phylogenetic tree. Like the CGTs from seed plants, the C-terminal of ScCGT1 also has the signature plant secondary product glycosyltransferase (PSPG) motif (Additional file 1: Fig. S4) [15]. However, different from the characterized CGTs in seed plants, the DPFXL motif conserved in 2-hydroxyflavanone CGTs was replaced by SHVLL in ScCGT1 (Additional file 1: Fig. S4) [15].

### Structural modeling and site-directed mutagenesis of ScCGT1

**Fig. 6:**
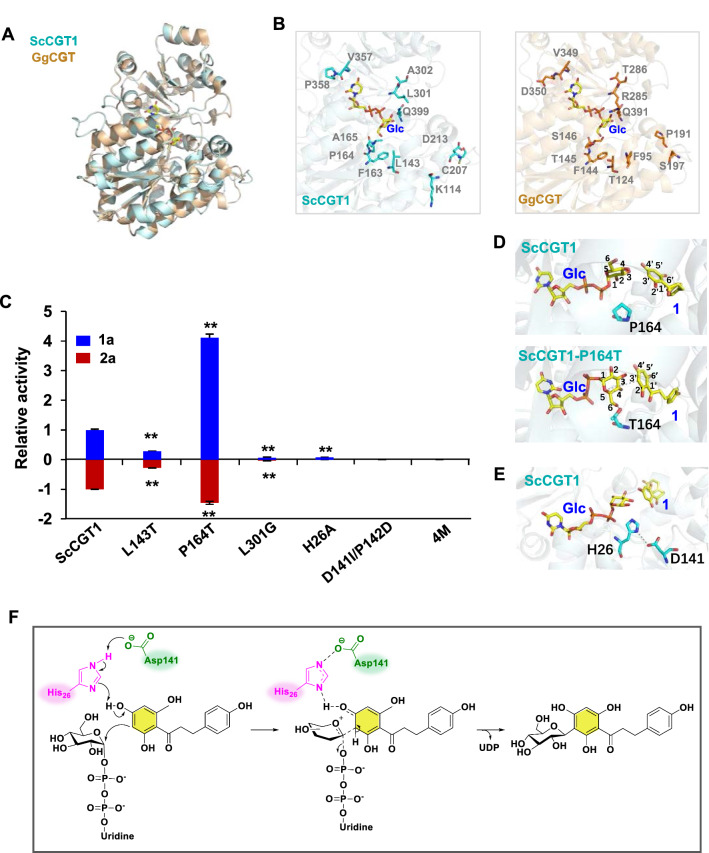
Structure-guided mutagenesis of ScCGT1. **A** Structure comparison analysis of ScCGT1 and GgCGT. **B** Comparison of the active sites of ScCGT1 and GgCGT. **C** The relative activities of wild-type ScCGT1 and mutants, using 1 and 2 as the substrates. The level of 1a and 2a from wild-type ScCGT1 was set as a value of 1.0. **D** Illustrations of docking of sugar donor (Glc) and sugar acceptor (phloretin) in the binding pocket of ScCGT1 and ScCGT1-P164T. **E** Location of His26 and Asp141 residues in the structure of ScCGT1. **F** A possible catalytic model for ScCGT1. Data presented in the form mean ± SD (n = 3); *p < 0.05 and **p < 0.01 (Glc, UDP-glucose; 1, phloretin; 2, 2-hydroxynaringenin)

The amino acids around the glycosyl donor (UDP-glucose) binding site (within 5 Å) are of great essence to the enzymatic activity of CGTs. Therefore, based on the results of the three-dimensional structure simulation and sequence alignment, the residues Leu143, Pro164, and Leu301 were identified and mutated to Thr (L143T), Thr (P164T), and Gly (L301G), respectively (Fig. 6A, B). For the substrates phloretin (1) or 2-hydroxynaringenin (2), the glycosylation activities of the mutants L143T and L301G were substantially decreased; while the activities from P164T were significantly higher than that of wild-type ScCGT1 (Fig. 6C). Compared with the structure of ScCGT1/UDP-Glc/phloretin (1), the C-3’ of phloretin in ScCGT1-P164T/UDP-Glc/phloretin (1) structure was closer to the anomeric carbon of UDP-Glc, which presumably was one of the reasons for facilitating this reaction (Fig. 6D). In addition, after Pro164 was mutated to Thr, the − OH group in Thr could form a hydrogen bond with the 6-OH of UDP-Glc. Therefore, we speculated that the Thr residue may play an important role in stabilizing the orientation of UDP-Glc in protein.

In previous studies, a His residue at the 5’-end (between 10–30 amino acids) was proved to act as a base, which could initiate the glycosylation reaction to generate *C*-glycoside by activating deprotonation [31]. This residue was located at the His26 site of ScCGT1. When His26 was mutated to Ala, the glycosylation activities decreased drastically (Fig. 6C). In addition, the D141I/P142D mutation also completely abolished *C*-glycosylation activity against phloretin (1) or 2-hydroxynaringenin (2) (Fig. 6C). Based on these results, we postulated that Asp141 may play a localizing role to stabilize the charge of His26 during the catalytic process (Fig. 6E). Therefore, the possible *C*-glycosylation process catalyzed by ScCGT1 was speculated (Fig. 6F). Initially, His26 acts as a base to receive a proton from the aromatic ring, which produced a positively charged intermediate. Then, the negatively charged Asp141 may stabilize the positively charged intermediate formed from the His26-substrate transition state. Thus, the catalytic dyad His26-Asp141 act cooperatively to facilitate the *C*-glycosylation of ScCGT1.

ScCGT1 does not contain the DPFXL motif which was considered a predictor for 2-hydroxyflavanone CGT activity in seed plants (Additional file 1: Fig. S4). Hence, SHVL (residues 108–111) was replaced with DPFF and the *C*-glycosylation activities of this quadruple variant (4 M) were abolished thoroughly (Fig. 6C). Although CGTs from ferns and seed plants have similar catalytic activity, their DPFXL motifs are different.

### Kinetic parameters of ScCGT1

Using phloretin (1) as the acceptor, ScCGT1 showed maximal activity at pH 6.5 (200 mM MES buffer) and 30 °C (Additional file 1: Fig. S5). Under the optimal reaction conditions, the kinetic properties of ScCGT1 with phloretin (1) and 2-hydroxynaringenin (2) were examined. The *K*_*m*_ and *k*_*cat*_/*K*_*m*_ values for phloretin (1) were 23.8 ± 2.8 μM and 559.8 M^−1^ s^−1^ (Table 1), respectively, and those for 2-hydroxynaringenin (2) were 17.5 ± 2.0 μM and 795.4 M^−1^ s^−1^ (Table 1). Consistent with the enzyme activity results, the P164T mutant produced *C*-glycosides with 2.5-fold improvements in catalytic efficiency (*k*_*cat*_/*K*_*m*_ = 1357.5 M^−1^ s^−1^) for phloretin (1) over the reaction catalyzed by wild-type ScCGT1 (Table 1). Meanwhile, the *k*_*cat*_/*K*_*m*_ value (1522.0 M^−1^ s^−1^) for 2-hydroxynaringenin (2) of P164T was also increased by more than two fold compared with ScCGT1 (Table 1; Additional file 1: Fig. S6).

**Table 1:** The kinetic parameters of recombinant ScCGT1 using phloretin (**1**) and 2-hydroxynaringenin (**2**) as substrate and UDP-glucose as the sugar donor

Enzyme	Substrate	*K*_*m*_ (μM)	*V*_*max*_ (nmol mg^−1^ min^−1^)	*k*_*cat*_ (min^−1^)	*k*_*cat*_/*K*_*m*_(M^−1^ s^−1^)
ScCGT1	Phl	23.8 ± 2.8	11.0 ± 0.3	0.8 ± 0.02	559.8
ScCGT1-P164T	Phl	29.8 ± 2.5	33.4 ± 0.7	2.4 ± 0.05	1357.5
ScCGT1	2-OHNA	17.5 ± 2.0	11.5 ± 0.3	0.8 ± 0.01	795.4
ScCGT1-P164T	2-OHNA	31.7 ± 2.9	39.9 ± 0.9	2.9 ± 0.06	1522.0

Phl, phloretin; 2-OHNA, 2-hydroxynaringenin

### Biotransformation of phloretin in *E. coli*

**Fig. 7:**
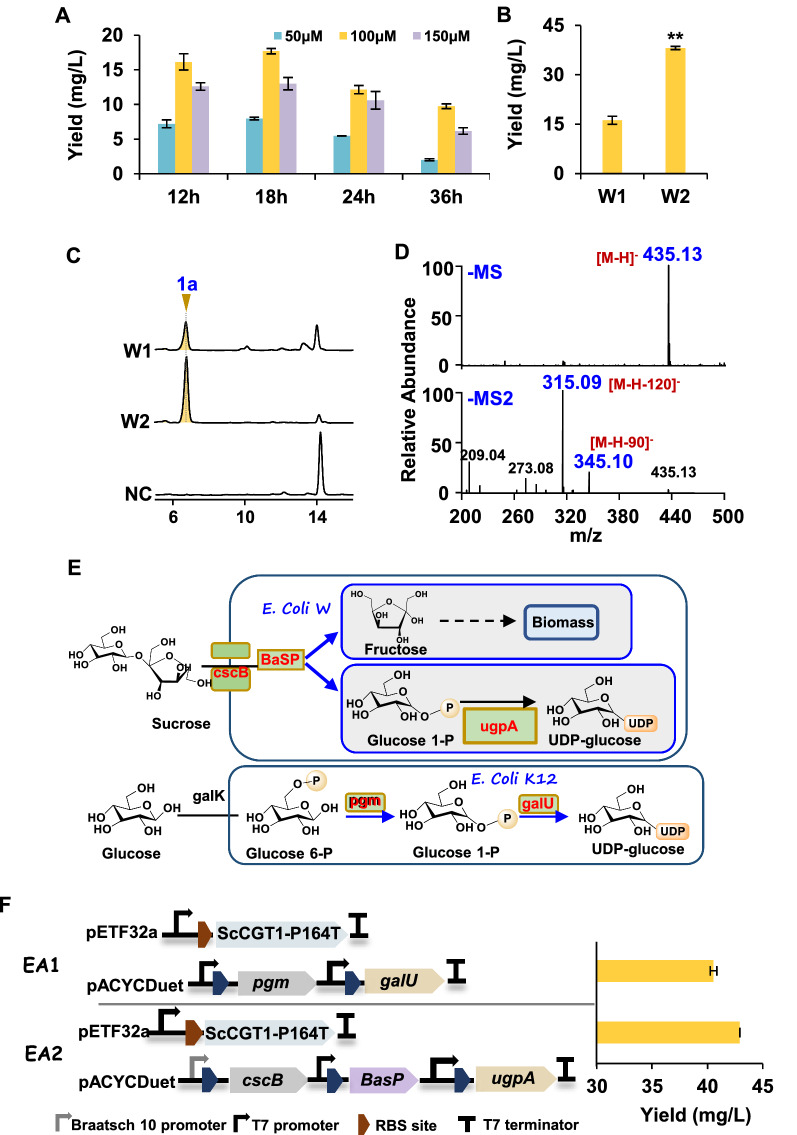
Production of nothofagin (1a) by bioconversion in *E. coli* cell. **A** The effect of phloretin (1) concentrations ranging from 50 to 150 μM on the production of 1a. **B** The production of 1a in *E. coli* strains W1 and W2. **C** HPLC analysis of the reaction products of *E. coli* strains W1, W2, and negative control (NC) fed with 100 μM phloretin (1). **D** Typical negative ion MS and MS2 spectra of product 1a. **E** The two alternative metabolic pathways are used for UDP-glucose synthesis. **F** *E. coli* EA1 and EA2 produced 1a yield when fed with 100 μM phloretin (1). Data presented in the form mean ± SD (n = 3); *p < 0.05 and **p < 0.01 (MS: mass spectrometry; MS2: tandem mass spectrometry)

To investigate the maximum bioconversion ability of *E. coli* strain W1 (harboring the ScCGT1 gene), varying concentrations of phloretin (1) were added to the culture media at final concentrations of 50, 100, and 150 μM. As described in Fig. 7A, the highest nothofagin (1a) production (17.7 mg/L) was detected under the 100 μM phloretin (1) with a corresponding molar conversion of 41%. In optimized reactions, nothofagin (1a) production in strain W2 (containing ScCGT1-P164T gene) was 38.4 mg/L and achieved high *C*-glycosylation conversion rates (> 80%) (Fig. 7B–D; Additional file 2: Table S3).

A reasonable supply of UDP-glucose in vivo was also important to improve the *C*-glycosylation reactions [32]. In previous reports, two exogenous UDP-glucose synthesis pathways were introduced in *E. coli* by constructing two plasmids (pACYCDuet-pgm-galU and pACYCDuet-cscB-BaSP-ugpA; Fig. 7E) [22, 33]. The effects of overexpression of UDP-glucose synthesis promoting protein on bioconversion of phloretin (1) were also evaluated by feeding 100 μM of the substrate in strains EA1 (harboring pACYCDuet-pgm-galU and pET32a-ScCGT1-P164T) and EA2 (consisting of pACYCDuet-cscB-BaSP-ugpA and pET32a-ScCGT1-P164T), respectively. Regarding nothofagin (1a) production, strain EA2 was found to be more efficient (about 43 mg/L, > 98% conversion) than strain EA1 (about 40 mg/L, > 92% conversion) (Fig. 7F; Additional file 2: Table S3).

### Production of vitexin and isovitexin in *E. coli*

**Fig. 8:**
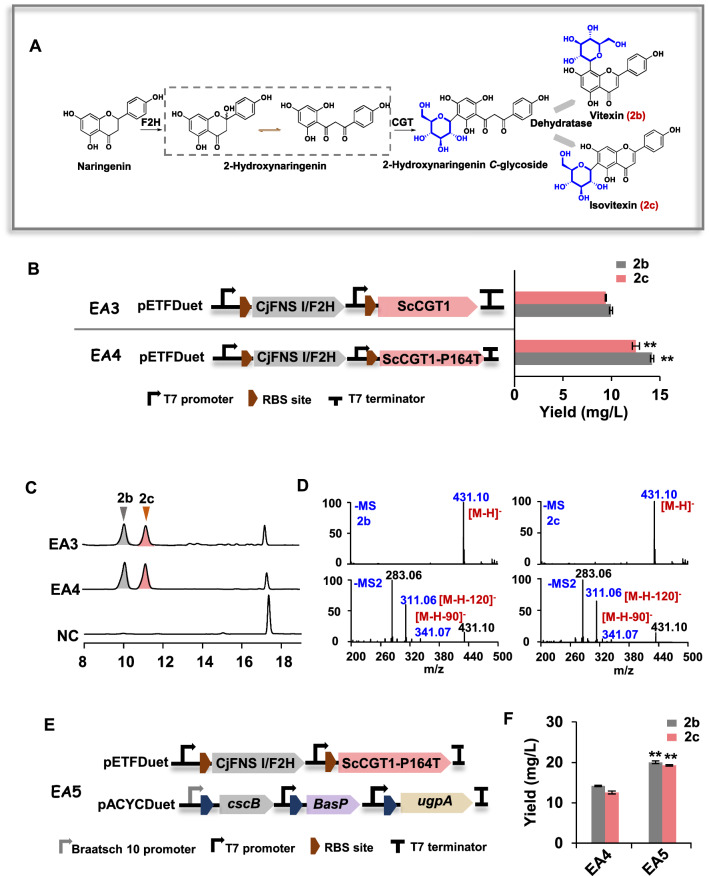
Production of vitexin (2b) and isovitexin (2c) by bioconversion in *E. coli* cell. **A** A simplified scheme showing the biosynthetic pathway for vitexin (2b) and isovitexin (2c). **B** The production of 2b and 2c in *E. coli* EA3 and EA4. **C** HPLC analysis of the reaction products of *E. coli* strains EA3, EA4, and negative control (NC) fed with 100 μM naringenin. **D** Typical negative ion MS and MS2 spectra of products 2b and 2c. **E** The genetic modules of strain EA5 in operon form. **F** The quantification of 2b and 2c produced by strains EA4 and EA5 fed with 100 μM naringenin. Data presented in the form mean ± SD (n = 3); *p < 0.05 and **p < 0.01 (MS: mass spectrometry; MS2: tandem mass spectrometry)

To test the ability of ScCGT1 to produce vitexin and isovitexin in vivo. CjFNS I/F2H from liverwort and ScCGT1 were co-expressed in *E. coli* (strain EA3). F2H enzymes introduced a hydroxy group at the C2 position of flavanones, and the resulting product then served as the substrate for the CGTs (Fig. 8A). So, the recombinant strain EA3 was first fed with various concentrations of naringenin (50, 100, and 150 μM) (Additional file 1: Fig. S7). With all three concentrations, EA3 gave the maximum conversion rate reached 44% when supplemented with 100 μM of naringenin, the yield reached around 10 mg/L vitexin and 9 mg/L of isovitexin. After replacing ScCGT1 with the ScCGT1-P164T gene (strain EA4), the yield of vitexin (14 mg/L) and isovitexin (13 mg/L) was increased by 40% and 44%, respectively (Fig. 8B). Representative HPLC chromatograms and product identification results are shown in Fig. 8C, D). When the sugar donor synthesis pathway was combined with CjFNS I/F2H-ScCGT1-P164T (strain EA5), significantly higher amounts of vitexin (20 mg/L) and isovitexin (19 mg/L) could be detected, and approximately 91% of the substrate was transformed (Fig. 8E, F); Additional file 2: Table S3).

### Subcellular localization of ScCGT1

To estimate the subcellular localizations of ScCGT1 protein, the full-length coding sequence of ScCGT1 was fused in-frame to a green fluorescent protein (GFP), then transiently expressed in *Nicotiana benthamiana* leaf. Confocal microscopy analysis demonstrated that the ScCGT1 protein is deposited in the nucleus and cytoplasm (Additional file 1: Fig. S8), which was consistent with the results from other plant glycosyltransferases [34, 35].

## Discussion

### Functional characterization of ScCGT1 from *S. chusanum*

The majority of characterized CGTs originate from plants, specifically cereals, legumes, citrus, and other plant species [11]. However, there are no reports about CGT in non-seed plants. *S. chusanum*, a traditional Chinese medicine, acts as a therapeutic remedy for various illnesses [36]. Vitexin and orientin, two important *C*-glycosides in *S. chusanum* [29], have beneficial effects on human health [9]. In this work, a 2-hydroxyflavanone CGT gene ScCGT1 was identified, which was involved in the biosynthesis of vitexin and orientin. Interestingly, although ScCGT1 accepts 2-hydroxyflavanones as substrate in vitro, phylogenetic tree analysis places ScCGT1 in a separate cluster with TcCGT1 and at the root of the CGT cluster (Fig. 5). ScCGT1 shares low level (18–28%) sequence homology with these previously reported plant CGTs. Moreover, a DPFXL motif was considered to predict the activity of seed plants 2-hydroxyflavanone CGTs [15], which were replaced by SHVLL in ScCGT1 (Additional file 1: Fig. S4). Although CGT from seed plants and ferns contained different active motif, they showed similar catalytic activity. Additionally, the substitution of SHVL in ScCGT1 (corresponding to DPFF) completely abolished the activities toward all the test substrates (Fig. 6C). These results indicated that CGT from ferns and seed plants had different catalytic amino acids motif. Structural modeling and site-directed mutagenesis data demonstrate that replacing Pro164 with Thr in ScCGT1 significantly improves *C*-glycosylation capacity (Fig. 6C; Table 1). P164T substitution may affect the binding of sugar donor and acceptor (Fig. 6D). This is similar to previous studies on CGT in *Scutellaria baicalensis*, *O. sativa*, *Z. mays*, *Arisaema erubescens*, *Pistia stratiotes*, *Landoltia punctata*, and *Glycyrrhiza uralensis* [37]. They explained the TA/TS motif stabilized the conformation of sugar donors through hydrogen bonding and hydrophobicity [37]. In both *O−* and *C*-glycosyltransferases, the N-terminal conserved His residue has been proved to initiate glycosylation by facilitating deprotonation [31]. In docking structure of ScCGT1/UDP-Glc/phloretin (1), His26 in ScCGT1 was far away from the phloretin (1) (Fig. 6E). However, the enzyme assays of mutant H26A illustrated that His26 may still function as a base to activate deprotonation. Asp residue act as a member of His-Asp dimer and has been studied extensively. For example, the Asp120-Ile121 active-site motif in OsCGT corresponding to Ile117-Asp118 in PcOGT (*O*-glycosyltransferase in *Pyrus communis*) was considered to be a switching site of *O*- and *C*-glycosylation functions. Compared with the wild enzyme, the obtained mutants (OsCGT or PcOGT) changed the specificity of glycosylation, but its activity also decreased dramatically [38]. These two amino acids were mapped to Asp141-Pro121 of ScCGT1. Different from the previous study [38], mutant D141I/P142D destroyed the *C*-glycosylation activity of ScCGT1. The reason was probably that Asp141 was closed to His26, and the negative charge of Asp141 might increase the proton-accepting capacity of His. The D141I/P142D mutation was accompanied by the disappearance of this interaction. Therefore, these two conserved residues of ScCGT1 synergistically promoted enzymatic *C*-glycosylation.

### Application of *C*-glycosyltransferases in biotechnology

At present, various *C*-glycosides sodium-dependent glucose cotransporter 2 (SGLT2) inhibitors were developed based on the phloretin *O*-glycoside (phlorizin). Nothofagin, the phloretin 3′-*C*-glycoside, has attracted considerable attention mainly due to its medicinal properties, including antioxidative activities [39] and as a potential therapeutic agent for renal disorders [40]. In this study, ScCGT1 exhibited *C*-glycosylation activity against phloretin, which was usually an important skeletal source for the synthesis of SGLT2 inhibitors. Site-directed mutation results illustrated that the P164T mutant stabilizes the whole structure and increases the activity of the *C*-glycosylation. The *K*_*m*_ value of ScCGT1-P164T was higher than several CGTs from seed plants, such as SbCGTa [38], while the *k*_*cat*_ was lower (Additional file 2: Table S4). However, of note, the recombinant *E. coli* expressing the ScCGT1-P164T showed a high *C*-glycosylation conversion rate (> 80%) for phloretin after 18 h fermentation (Fig. 7), which might render it a promising enzyme for designing various SGLT2 inhibitors. Furthermore, when UDP-glucose was supplied in vivo, the maximum conversion rate of ScCGT1-P164T toward phloretin reached 99% (about 43 mg/L; Additional file 2: Table S3), and the whole fermentation process only took 18 h.

The research on introducing plant F2Hs and CGTs into *E. coli* to produce flavone *C*-glycosides are extremely limited. At present, only two relevant studies have been reported, and both of them take a truncated F2H gene (in the transmembrane region) coupled with a P450 reductase (CPR) to co-express in *E. coli,* which provide the reaction intermediates for the catalysis of CGTs [18, 19]. In this study, the soluble protein CjFNS I/F2H and ScCGT1-P164T were combined in *E. coli*, which led to a high conversion from naringenin to vitexin (20 mg/L) and isovitexin (19 mg/L) (Fig. 8). The yields were similar to that previously reported [19, 20]. For example, Chen *et al*. constructed a recombinant *E. coli* by introducing two P450 genes (F2H and CPR) and one CGT gene, which resulted in 26.6 mg/L of isoorientin and 23.9 mg/L of orientin [19]. F2H acts as a rate-determining step that directly affects the synthesis efficiency of CGT in vivo [17, 41]. Unlike P450, CjFNS I/F2H is a soluble 2-ODD enzyme that does not require CPR for effective electron transfer in the process of catalytic hydroxylation. However, CjFNS I/F2H possesses both F2H and FNS I activities. It catalyzed naringenin to 2-hydroxynaringenin and a considerable amount of apigenin, which limited supply of the substrate 2-hydroxynaringenin for the production of *C*-glycosides. Therefore, CjFNS I/F2H will be engineered by directed evolution with enhanced chemo- and regio-selectivity to increase the 2-hydroxynaringenin production.

## Conclusion

Taken together, ScCGT1 represents the first CGT identified from ferns. Homology-based protein modeling and site-directed mutagenesis indicated that P164T substitution exhibited a robust *C*-glycosylation capacity toward phloretin and 2-hydroxynaringenin in vitro, which have increased about 1.5–4 folds compared with ScCGT1. It might be exploited as a biocatalyst to efficiently generate nothofagin in vivo. Moreover, coexpression of CjFNS I/F2H and ScCGT1 in *E.coli* resulted in the production of flavone *C*-glycosides. The current study provides a candidate gene and strains for efficient synthesis of high-value *C*-glycosides (nothofagin, vitexin and isovitexin) and offers an enzymatic tool for metabolic engineering of phytochemicals with health benefits.

## Materials and methods

### Plant materials and reagents

*Stenoloma chusanum* was collected from Fujian (23°54′N, 118°379′E), Guangdong (23°72′N,113°04′E), Guizhou (25°19′N, 107°17′E), Yunnan (25°11′N, 99°17′E), Hunan (26°22′N, 111°63′E), Zhejiang (28°66′N, 121°42′E), and Guangxi (22°64′N, 110°14′E), respectively. Leaves, roots, and stems were collected. *N. benthamiana* were grown under normal conditions with a light/dark photoperiod of 16-h/8-h at a temperature of 25 °C.

Naringenin, eriodictyol, phloretin, vitexin, isovitexin, orientin, and isoorientin were obtained from Chengdu Must Biotechnology (Chengdu, China). The UDP-glucose, UDP-galactose, and UDP- glucuronic acid were purchased from Sigma-Aldrich (St. Louis, USA). 2-hydroxynaringenin and 2-hydroxyeriodictyol were synthesized by a plant CjFNS I/F2H following a published procedure [42].

### Analysis of flavonoids in *S. chusanum*

The freeze-dried plant tissues were ground in 600 μL 80% (v/v) methanol (100 μM chrysin was used as an internal standard), and sonicated for 1 h at room temperature. After centrifugation at 6,000 g for 20 min, the extracts were filtered through a 0.2 μm microporous filter for HPLC analysis. The samples were separated by a 5-μm reverse-phase XDB-C18 column (Phenomenex Luna). The mobile phase was a gradient elution of 0.1% (v/v) formic acid water (A) and acetonitrile (B). HPLC elution program was as follows: 0 to 45 min, 15% to 30% (v/v) B; 45 to 50 min, 30% to 45% (v/v) B; 50 to 55 min, 45% to 100% B, with a flow rate of 0.8 mL/min and an injection volume of 20 μL.

### CGTs identification and quantitative real-time PCR analysis

The putative genes were identified by searching the Swissport databases of *S. chusanum* (SRR8185333) using the keyword of UDP-glycosyltransferase. Combined with the blast analysis, four candidate genes were selected for further functional characterization. Accumulation of ScGTs transcripts was measured by quantitative real-time PCR (qRT-PCR) with an Eppendorf realplex2 system using TB Green TM Premix Ex Taq TM (TliRnaseH Plus, Takara, China). The primers were designed using BioXM software (version 2.6). An *ACTIN* gene was used as an internal control, and each data were calculated from three biological replicates. The program of qPCR analysis was performed as Niu *et al*. described [43]. The relative expression levels of the target genes were evaluated using the 2^−ΔΔC(T)^ method (the related primers were given in Additional file 2: Table S5).

### Molecular cloning and expression of UGT genes from *S. chusanum*

The total RNA of *S. chusanum* leaves was prepared using the cetyltrimethylammonium bromide (CTAB)-based procedure. Then reverse-transcribed to cDNA with a ReverTra Ace® qPCR RT kit (ToYoBo, Osaka, Japan) following the manufacturer’s instructions. The candidate genes were amplified by PCR with gene-specific primer pairs (Additional file 2: Table S6) using Ape X *HF* FS PCR Master Mix (Accurate Biology, China). Then the candidate CGTs were subcloned into pET32a (+) vectors (Novagen, USA) (primer pairs listed in Additional file 2: Table S7). The recombinant proteins were expressed in *E. coli* strain BL21 (DE3) and extracted in accordance with the previous report [44].

### Glycosyltransferase activity assays

The enzyme assay was performed with 200 mM Tris HCl (pH 7.5), 1 mM Dithiothreitol (DTT), 0.1 mM substrate (phloretin, apigenin, luteolin, 2-hydroxynaringenin, and 2-hydroxyeriodictyol), 1 mM sugar donor, and 10 μg of purified proteins (35 °C, 1 h) [34]. The reactions were terminated with methanol or HCl. Protein harboring empty pET32a plasmid was used for control assays. After centrifugation (8,000 g for 30 min), the supernatants were collected and analyzed by HPLC and LC/MS, equipped with an Agilent reverse-phase XDB-C18 column (250 mm × 4.6 mm, 5 μm) at a flow rate of 0.8 ml min^−1^. The mobile phase consisted of two eluents: (A) 0.1% (by volume) aqueous acetic acid and (B) methanol. For the products formed from phloretin, apigenin, or luteolin, the HPLC elution program was as follows: 0 min, 35% B; 20 min, 65% B. When the 2-hydroxyflavaones were provided as the substrates and methanol was used to terminate the reaction, the HPLC elution program was as follows: 0 min, 25% B; 10 min, 35% B; 20 min, 80% B. In the quenching reaction with HCl, the initial B concentration was 30%, which increased to 45% after 10 min, followed by a linear gradient from 45 to 80% B for 10 min.

### Determination of kinetic parameters

To assay for the optimal reaction temperature of ScCGT1, the enzymatic reactions were carried out at pH 7.5 with different temperatures (20–60 °C). Then, the enzymatic reactions were incubated at 30 °C in various reaction buffers ranging in pH values from 5.0–6.5 (MES buffer), 6.5–7.5 (Tirs-HCl buffer), and 7.5–9.0 (K_2_HPO_4_-KH_2_PO_4_ buffer).

For the kinetic assay of ScCGT1, the reaction was carried out at 30 °C for 15 min. Briefly, an enzymatic assay containing 200 mM MES buffer (pH 6.5), 5 μg of purified ScCGT1, 1 mM UDP-Glc, and various concentrations of phloretin or 2-hydroxynaringenin (5, 10, 20, 50, 75, 100, 150, 200, and 300 μM). All the reactions were quenched with an equal volume of methanol. Kinetic parameters of the enzyme reaction were obtained by fitting the kinetics data to the Lineweaver–Burk plots using the GraphPad Prism 5 software.

### Bioinformatics analysis

Multiple sequence alignment was implemented using DNAMAN v7.0.2 software (Lynnon Biosoft, Quebec, Canada) [45]. A phylogenetic tree was constructed using CLUSTAL W implemented in MEGA v.4.0 based on the neighbor-joining method [46] with the following parameters: bootstrap method (1000 replicates), *p*-distance model, uniform rates, and pairwise deletion.

### Structural modeling and site-directed mutagenesis

The structural model of ScCGT1 was obtained from the SWISS-MODEL server [47] based on the crystal structure of GgCGT (PDB ID: 6L5P) [28]. The sugar donor and acceptor were inserted into the model using the Schrodinger Suites program (www.schrodinger.com). All models were generated using PyMOL.

Site-directed mutagenesis of ScCGT1, including H26A, L143T, P164T, L301G, D141I/P142D, and S108D/H109P/V110F/L111F (named 4 M) mutants, were constructed using the Stratagene QuickChange (ABclonal Technology, China) site-directed mutagenesis method [48] (The necessary primer pairs were listed in Additional file 2: Table S8). Enzyme assays of the mutated proteins were performed under the same conditions as described above.

### Plasmid construction and *C*-glycosides production by recombinant strains

The complete set of plasmids and strains used in this study are listed in Table 2 (the necessary primers were given in Additional file 2: Table S9). *E. coli* cells harboring the sequences of ScCGT1 and ScCGT1-P164T were named W1 and W2, respectively. The plasmids pET32a-ScCGT1-P164T and pACYCDuet-Pgm-GalU [32] were transformed into *E. coli* BL21 (DE3) to construct the recombinant strain EA1. Similarly, EA2 was obtained by introducing both pET32a-ScCGT1-P164T and pACYCDuet-cscB-Basp-UgpA [32]. CjFNS I/F2H and ScCGT1 were ligated into the pETDuet-1 vector (Novagen, USA) under *Bam*H I/*Hin*d III sites and *Bgl* II/*Kpn* I sites to create pETDuet-1-CjFNS I/F2H-ScCGT1, which was transformed into *E. coli* BL21 (DE3) cells (named strain EA3). In a similar way, strain EA4 containing CjFNS I/F2H and ScCGT1-P164T genes was constructed. The plasmids pETDuet-1-CjFNS I/F2H-ScCGT1-P164T and pACYCDuet-cscB-Basp-UgpA were used for the co-transformation of *E. coli* BL21 (DE3) cells to construct the recombinant strain EA5.

**Table 2:** Plasmids and strains used in this study

Plasmids or strains	Genotype	Source
Plasmids		
pETDuet-1	Double T7 promoters, pBR322 ori, Amp^R^	Novagen
pET32a (+)	T7 promoter, F1 ori, Amp^R^	Novagen
pET32a-ScCGT1	pET32a-ScCGT1	This study
pET32a-ScCGT1-P164T	pET32a-ScCGT1-P164T	This study
pETDuet-1-CjFNS I/F2H	pETDuet-1 carrying CjFNS I/F2H from *Conocephalum japonicum*	This study
pETDuet-1-CjFNS I/F2H-ScCGT1	pETDuet-1 carrying CjFNS I/F2H from *C. japonicum* and ScCGT1	This study
pETDuet-1-CjFNS I/F2H-ScCGT1-P164T	pETDuet-1 carrying CjFNS I/F2H from *C. japonicum* and ScCGT1-P164T	This study
pACYCDuet-*cscB*-*Basp*-*UgpA*	pACYCDuet carrying *cscB* from *E. coli* W, *Basp* from *Bifidobacterium adolescentis*, and *ugpA* from *B. bifidum*	[32]
pACYCDuet-*Pgm*-*GalU*	pACYCDuet carrying *pgm* and *galU* from *E. coli* K12	[32]
Strains		
*E. coli* DH5α		Takara
*E. coli* BL21		Takara
W1	BL21(DE3) carrying pET32a-ScCGT1	This study
W2	BL21(DE3) carrying pET32a-ScCGT1-P164T	This study
EA1	BL21(DE3) carrying pET32a-ScCGT1 and pACYCDuet-*Pgm*-*GalU*	This study
EA2	BL21(DE3) carrying pET32a-ScCGT1-P164T and pACYCDuet-*cscB*-*Basp*-*UgpA*	This study
EA3	BL21(DE3) carrying pETDuet-1-CjFNS I/F2H-ScCGT1	This study
EA4	BL21(DE3) carrying pETDuet-1-CjFNS I/F2H-ScCGT1-P164T	This study
EA5	BL21(DE3) carrying pETDuet-1-CjFNS I/F2H-ScCGT1-P164T and pACYCDuet-*cscB*-*Basp*-*UgpA*	This study

Recombinant strains precultures were grown in a 3 mL LB medium containing appropriate antibiotics (100 μg/mL ampicillin or 30 μg/mL chloramphenicol). Seed culture (100 μL) was injected into 10 mL of fresh LB medium. Cultures were grown at 37 °C with 110 rpm orbital shaking. After the OD600 reached 0.5–0.8, the isopropyl-*β*-D-thiogalactopyranoside (IPTG) was added at a final concentration of 0.5 mM, then incubated further at 16 °C for 6 h. The cultures were then supplemented with different concentrations (50, 100, and 150 μM) of substrate (phloretin or naringenin) and incubated at 16 °C and 110 rpm for 36 h.

Aliquots of the cultures were taken after 12, 18, 24, and 36 h culture and extracted twice with the equivalent volume of n-butyl alcohol. The combined supernatant was evaporated and finally dissolved in 100 μL of 80% (v/v) methanol for HPLC analysis. Sub-cellular localization of ScCGT1.

ScCGT1 was amplified with primers ScCGT1-GFP-F/GFP-R (Additional file 2: Table S10) for C-terminal GFP fusion; the amplified DNA fragment was introduced into the Gateway Entry vector pDONR 207 (Invitrogen, Carlsbad, USA) using the BP Clonase reaction. After being confirmed by sequencing, the ScCGT1 entry vectors were then subcloned by LR and recombined into the binary vector pGWB5 (Invitrogen, Carlsbad, USA). Finally, the localization constructs were transformed into *Agrobacterium tumefaciens* GV3101 cells. For transient expression analysis, the ScCGT1 correct construct was infiltrated into the bottom of *N. benthamiana* leaves. GFP signals were detected following the procedure reported previously [34].

## Supplementary Information

**Additional file 1: Fig. S1. **The possible biosynthetic pathways for *C*-glycosylflavones. F2H: flavanone 2-hydroxylase; FNS: flavone synthase; CGT: *C*-glycosyltransferase.** Fig. S2. **SDS-PAGE analysis of recombinant proteins. M: Weight marker, Lane 1: pET32a, Lane 2: ScGT1, Lane 3: ScGT2, Lane 4: ScGT3, Lane 5: ScGT4, Lane 6: ScCGT1-H26A, Lane 7: ScCGT1-L143T, Lane 8: ScCGT1-P164T, Lane 9: ScCGT1-L301G, Lane 10: ScCGT1-D141I/P142D, Lane 11: ScCGT1-4T (S108D/H109P/V110F/L111F).** Fig. S3.** Sugar donor selectivity of ScCGT1. A. HPLC chromatograms of the glycosylation reactions using UDP-galactose as sugar donor. B. Typical negative ion MS and MS2 spectra of product 1b. C. HPLC chromatograms of the glycosylation reactions using UDP-glucuronic acid as sugar donor. D. Structures of the sugar donors. E. Relative activity of glycosylated products (1a and 1b) using three sugar donors. Phloretin was used as the acceptor substrate (1, phloretin; Gal, UDP-galactose; GlcA, UDP-glucuronic acid; Glc, UDP-glucose; MS:mass spectrometry; MS2: tandem mass spectrometry).** Fig. S4.** Sequence alignment of ScCGT1 with CGTs from other plants. The UGTs’ signature PSPG motifs were enclosed in a red box. The abbreviations for species and accession numbers are listed in Additional file 2: Table S2. **Fig. S5.** Enzymatic characteristics of purified recombinant ScCGT1. Effect of various pH (A) and temperature (B) on the enzyme activities of ScCGT1 using phloretin as substrates, UDP-glucose as sugar donor.** Fig. S6.** Kinetic analysis of ScCGT1 and ScCGT1-P164T mutant. (1, phloretin; 2, 2-hydroxynaringenin; Glc, UDP-glucose). **Fig. S7. **The effect of varying the concentration of naringenin on the production of vitexin (2b) and isovitexin (2c).** Fig. S8.** Subcellular localization of *ScCGT1*. Sub-cellular localization of vector pGWB5 and the products of the transgenes ScCGT1-GFP in transiently transformed tobacco leaf discs. The GFP signal appears green and the chlorophyll signal is red.

**Additional file 2: Table S1. **The sequences of UGT genes in *S. chusanum*. **Table S2.** Accession numbers of amino acid sequences used for glycosyltransferases phylogenetic reconstruction.** Table S3.** The conversion rate generated by the phloretin or naringenin to produce the corresponding *C*-glycosides at the 100 μM substrate concentration.** Table S4. **The kinetic parameters of recombinant ScCGT1 and other CGTs using phloretin (1) or 2-hydroxynaringenin (2) as substrate and UDP-glucose as the sugar donor. **Table S5.** The primers were used for qRT-PCR analysis.** Table S6.** The primers were used to obtain the full length of ScGTs genes.** Table S7. **Sequences of specific primers for PCR.** Table S8.** The primers were used for the site-directed mutagenesis vector construction.** Table S9.** The primers used for plasmid constructions (into pETDuet-1 vector).** Table S10. **Primers for sub-cellular localization analysis.

## Data Availability

All data for this study are included in this published article and its additional file.
