# Supplementary material for: Identification of a flavonoid C-glycosyltransferase from fern species Stenoloma chusanum and the application in synthesizing flavonoid C-glycosides in Escherichia coli
Source: Microb Cell Fact. 2022 Oct 14;21:210. doi: 10.1186/s12934-022-01940-z (PMC9563126; doi:10.1186/s12934-022-01940-z)
Supplement: Supplementary file 1 — Additional file 1: Fig. S1. The possible biosynthetic pathways for C-glycosylflavones. F2H: flavanone 2-hydroxylase; FNS: flavone synthase; CGT: C-glycosyltransferase. Fig. S2. SDS-PAGE analysis of recombinant proteins. M: Weight marker, Lane 1: pET32a, Lane 2: ScGT1, Lane 3: ScGT2, Lane 4: ScGT3, Lane 5: ScGT4, Lane 6: ScCGT1-H26A, Lane 7: ScCGT1-L143T, Lane 8: ScCGT1-P164T, Lane 9: ScCGT1-L301G, Lane 10: ScCGT1-D141I/P142D, Lane 11: ScCGT1-4T (S108D/H109P/V110F/L111F). Fig. S3. Sugar donor selectivity of ScCGT1. A. HPLC chromatograms of the glycosylation reactions using UDP-galactose as sugar donor. B. Typical negative ion MS and MS2 spectra of product 1b. C. HPLC chromatograms of the glycosylation reactions using UDP-glucuronic acid as sugar donor. D. Structures of the sugar donors. E. Relative activity of glycosylated products (1a and 1b) using three sugar donors. Phloretin was used as the acceptor substrate (1, phloretin; Gal, UDP-galactose; GlcA, UDP-glucuronic acid; Glc, UDP-glucose; MS:mass spectrometry; MS2: tandem mass spectrometry). Fig. S4. Sequence alignment of ScCGT1 with CGTs from other plants. The UGTs’ signature PSPG motifs were enclosed in a red box. The abbreviations for species and accession numbers are listed in Additional file 2: Table S2. Fig. S5. Enzymatic characteristics of purified recombinant ScCGT1. Effect of various pH (A) and temperature (B) on the enzyme activities of ScCGT1 using phloretin as substrates, UDP-glucose as sugar donor. Fig. S6. Kinetic analysis of ScCGT1 and ScCGT1-P164T mutant. (1, phloretin; 2, 2-hydroxynaringenin; Glc, UDP-glucose). Fig. S7. The effect of varying the concentration of naringenin on the production of vitexin (2b) and isovitexin (2c). Fig. S8. Subcellular localization of ScCGT1. Sub-cellular localization of vector pGWB5 and the products of the transgenes ScCGT1-GFP in transiently transformed tobacco leaf discs. The GFP signal appears green and the chlorophyll signal is red. [file 12934_2022_1940_MOESM1_ESM.pdf]

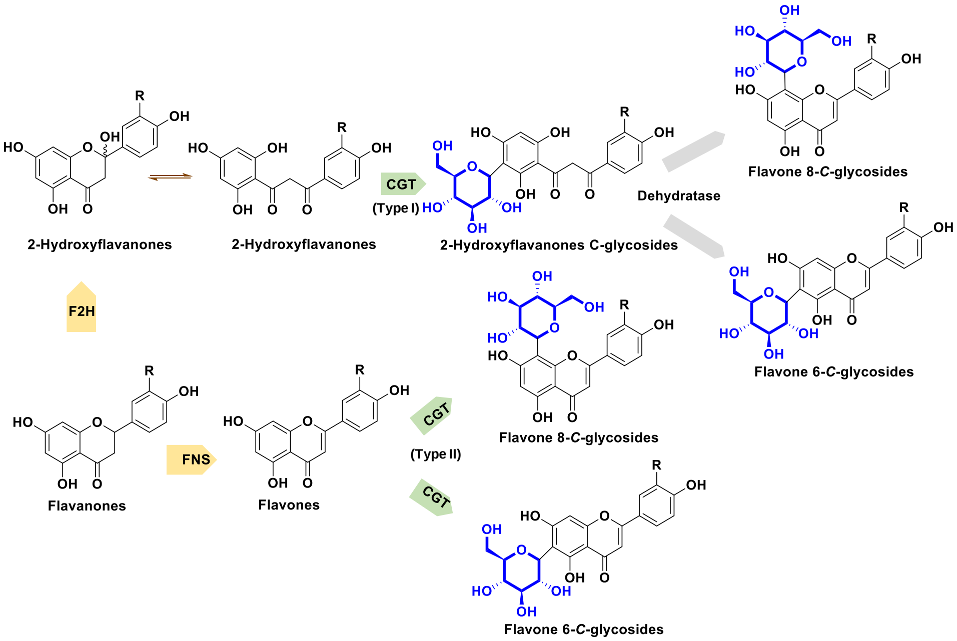

Fig. S1 The possible biosynthetic pathways for *C*-glycosylflavones. F2H: flavanone 2-hydroxylase; FNS: flavone synthase; CGT: *C*-glycosyltransferase.

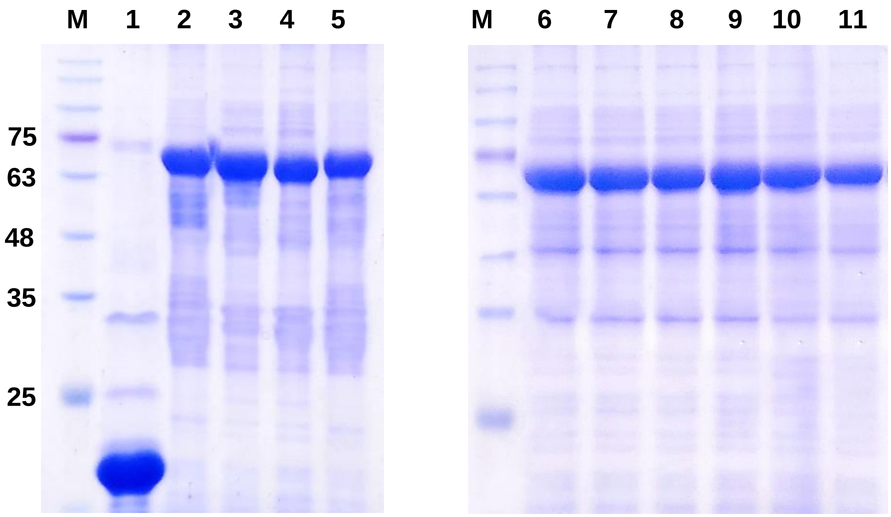

Fig. S2 SDS-PAGE analysis of recombinant proteins. M: Weight marker, Lane 1: pET32a, Lane 2: ScGT1, Lane 3: ScGT2, Lane 4: ScGT3, Lane 5: ScGT4, Lane 6: ScCGT1-H26A, Lane 7: ScCGT1-L143T, Lane 8: ScCGT1-P164T, Lane 9: ScCGT1-L301G, Lane 10: ScCGT1-D141I/P142D, Lane 11: ScCGT1-4T (S108D/H109P/V110F/L111F).

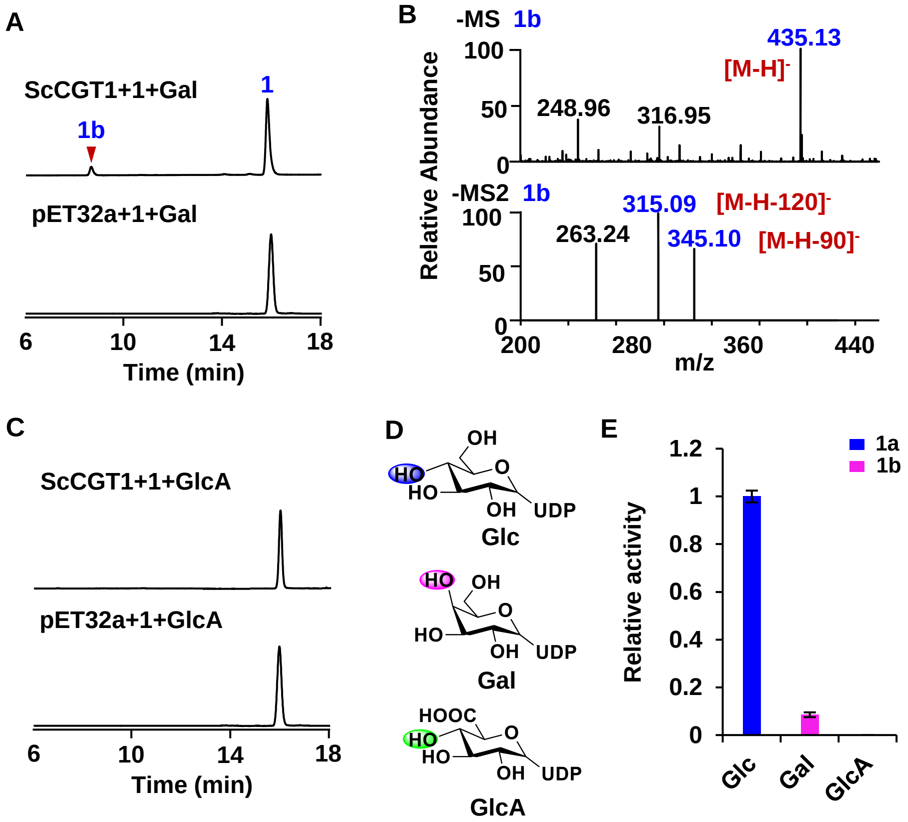

Fig. S3 Sugar donor selectivity of ScCGT1. A. HPLC chromatograms of the glycosylation reactions using UDP-galactose as sugar donor. B. Typical negative ion MS and MS2 spectra of product **1b**. C. HPLC chromatograms of the glycosylation reactions using UDP-glucuronic acid as sugar donor. D. Structures of the sugar donors. E. Relative activity of glycosylated products (**1a** and **1b**) using three sugar donors. Phloretin was used as the acceptor substrate (**1,** phloretin; Gal, UDP-galactose; GlcA, UDP-glucuronic acid; Glc, UDP-glucose; MS: mass spectrometry; MS2: tandem mass spectrometry).

```
ScCGT1   .MASCDTGLLSDQRHHALMFPYPVQGHIQPFMRLSKLLATNYGFTISFFNSEENHRRLLADRDEPASQEDRAEDA    74
TcCGT1   ...MEKSNPNSTSKPHVFLLASPGMGHLIPFLELSKRLVT.................LNTLQVTLFIVSNEATKA    55
SbCGTa   ...MASTTKSENVGAHIALFPCAGMGHLLPFLRLAAMLDA.................RGCA.VTVITVKPTVSAA    54
OsCGT    ...MPSSGDAAGRRPHVVLIPSAGMGHLVPFGRLAVALSS.................GHGCDVSLVTVLPTVSTA    55
ZmCGT    ..MAANGGDHTSARPHVVLLPSAGMGHLVPFARLAVALSE.................GHGCNVSVAAVQPTVSSA    56
GgCGT    MGENNMSSIAPHPVVHVALLPSAGMGHLTPFLRLASLLLH.................QHCH.VTLITPQPTVSKA    57

ScCGT1   EGK.PLDFRMLFLPDVLPSSTEVEQMSAGTHIEKSHVLLSKTFLNTDPAALETLIRSQSPP..ITCLILDPLVTV   146
TcCGT1   RSH.LMESSNNFHPDLELVDLTPANLSELLSTDATVFKRIFLITQAAIKDLESRISSMSTP..PAALIVDVFSMD   127
SbCGTa   ESDHLSAFFT.IHPRITRLEFQLLPYQKSGLRNDDPFFIQMETIATSVH.LLRPLLSSLSPPLSAIVSDFTLTSQ   127
OsCGT    ESKHLDALFD.AFPAVRRLDFELAPFDASEFPGADPFFLRFEAMRRSAP.LLGPLLTGAGA.SALATDIALTSV   126
ZmCGT    ESRLLDALFVAAAPAVRRLDFRLAPFDESEFPGADPFFLRFEATRRSAP.LLGPLLDAAEA.SALVTDIVLASV   128
GgCGT    EEDLLSRFLS.AFPQVNQLHFHLPPSDSTIST..DPFFLQFASIRSSSH.LLTPLLSSLTPPLSSFIYDMTLISP   128
```

DPXFL motif

```
ScCGT1   IAPGLGEKLGITSLIFFPASAASLATAIRSGEVDG.........DAKQMTRIVG..APVLDVKEVNPFL...SIC   207
TcCGT1   .AFPVADRFGIKKYVFVTLNAWFLALTTYVRTLDREIE..GEYVDLPEPIAIPG..CKPLRPEDVFDPMLSRSSD   197
SbCGTa   .VTDLVSDLPISTYTLMTSSAAFFCLMAYLPKLLQIDVAN....RDA....IEIPDLGPISMSSIPPKMLDPSDF   193
OsCGT    .VIPVAKEQGLPCHILFTASAAMLSLCAYFPTYLDANAGG...GGGVGDVDIPG..VYRIPKASIPQALHDPNHL   195
ZmCGT    .ALPVARERGVPCYVLFTSSAAMLSLCAYFPAYLDAHAAAGSVGVGVGNVDIPG..VFRIPKSSVPQALHDPDHL   200
GgCGT    .LLPIAESLGVPHYILFTSSATMFSFFSYFPTLAKSESFP....GKLDFVEIPGVSVSSIPRSSIPPPLLVPNSL   198

ScCGT1   GRGHSDFMARLFTQPFRKKLIKANDIILINTVEELEESTLKHLQEENKVCAIG.PVLPPWGAFAVPTLWSQDYS.   280
TcCGT1   GYRPYLGMSERLTKA.........DGLLLNTWEALEPVSLKALRENEKLNQIMTPPLYPVGPVARTTVQEVVG.N   262
SbCGTa   FSAFISSNVSSIHKV.........KGVLINTFNSFESEAIEAVRRNG..VDHILPIGPL........ESYDAKK   248
OsCGT    FTRQFVANGRSLTSA.........AGILVNTFDALEPEAVAALQQGK..VASGFPPVFAVGPLLPASNQAKDPQA   259
ZmCGT    FTQQFVANGRCLVAC.........DGILVNTFDAFEPDAVTALRQGSITVSGGFPPVFTVGPMLPVRFQAEET.A   265
GgCGT    FGKLFMEDSPKLKKL.........HGVLVNTFEGIEKLSLEALNGGK..VVKGLPPVYGVGPFVPCEFEKVVKRG   262

ScCGT1   .CL.DWLDKQPAASVLFVSFGSLATLSSTQLSDIALGLEASNQRILWVTRPDQIY..............GKAPDL   339
TcCGT1   ECL.DWLSKQPTESVLYVALGSGGIISYKQMTEIAWGLEMSRQRFIWVVRLPTMEKDGACRFFSDVNVKGPLEYL   336
SbCGTa   AHDLPWLDEQPPESVLFVSFGSRTALSKEQIRELGAALEKSGCRFLWVLKGGKVDKE.......DKEEVEDM..L   314
OsCGT    NYM.EWLDAQPARSVVYVSFGSRKAISREQLRELAAGLEGSGHRFLWVVKSTVVDRD.......DAAELGEL..L   324
ZmCGT    DYM.RWLSAQPPRSVVYVSFGSRKAIPRDQLRELAAGLEASGKRFLWVVKSTIVDRD.......DTADLGGL..L   330
GgCGT    ETISEWLDEQPSGSVVYVSFGSRTAMGREQLREVGDGLVKSGWRFLWVVKDKIVDRA.......EEEGLDGV..L   328

ScCGT1   PSDFLERI..KDRILVFS.WVPQLHVLCHTSVGGFLSHCGWNSTIESIAAGVPILAWPFFGDQMLNAKCVV..EK   409
TcCGT1   PEGFLDRN..KELGMVLPNWGPQDAILAHPSTGGFLSHCGWNSSLESIVNGVPVIAWPLYAEQKMNATLLT....   405
SbCGTa   GASFVERTK..KKGLIVKGWVKQEQILAHPAIGGFVSHCGWNSVIEAARLGVPVLAWPQHGDQSVNAGVVEKAGL   387
OsCGT    DEGFLERV..EKRGLVTKAWVDQEEVLKHESVALFVSHCGWNSVTEAAASGVPVLALPRFGDQRVNSGVVARAGL   397
ZmCGT    GDGFLERV..QGRAFVTMGWVEQEEILQHGSVGLFISHCGWNSLTEAAAFGVPVLAWPRFGDQRVNAALVARSGL   403
GgCGT    GFELVERMVKEKKGLVVKEWVDQSEILGHKAVGGFVSHCGWNSVVEAAWFGVKILGWPLHGDQKINAEVVAKGGW   403
```

PSPG motif

```
ScCGT1   W.........RIGLALTGAGGAMSKAVVETRVKDLMEGDLSKELRNRAQNLKHIVVKALSHGGSSSSNLQKLFGH   475
TcCGT1   ...EELGVAVRPEVLPTKAVV..SRDEIEKMVRRVIESKEGKMKRNRARSVQSDALKAIEKGGSSYNTLIEVAKE   475
SbCGTa   GLWVREWGWGQTKLIGREEIA...EKMIEVMQDEKLRVS........AGEVRAKAKETREVDGDSEALLQRLIHS   451
OsCGT    GVWADTWSWEGEAGVIGAEEI.SEKVKAAMADEALRMK........AASLAEAAKAVAGGGSSHRCLAEFARL   462
ZmCGT    GAWEEGWTWDGEEGLTTRKEV..AKKIKGMMGYDAVAEK........AAKVGDAAAAAIAKCGTSYQSLEEFVQR   468
GgCGT    GVWKEGWDWEGERLVKGEEIG...EAIREVMNDESLVMK........ATQVKKDARKAISVGGGCEVALQKLMEV   467

ScCGT1   L.......                                                                      476
TcCGT1   FEKNHKVL                                                                      483
SbCGTa   FNNITQNS                                                                      459
OsCGT    CQGGTCRT                                                                      470
ZmCGT    CRDAERK.                                                                      475
GgCGT    WKKNV...                                                                      472
```

Fig. S4 Sequence alignment of ScCGT1 with CGTs from other plants. The UGTs' signature PSPG motifs were enclosed in a red box. The abbreviations for species and accession numbers are listed in **Additional file 2: Table S10**.

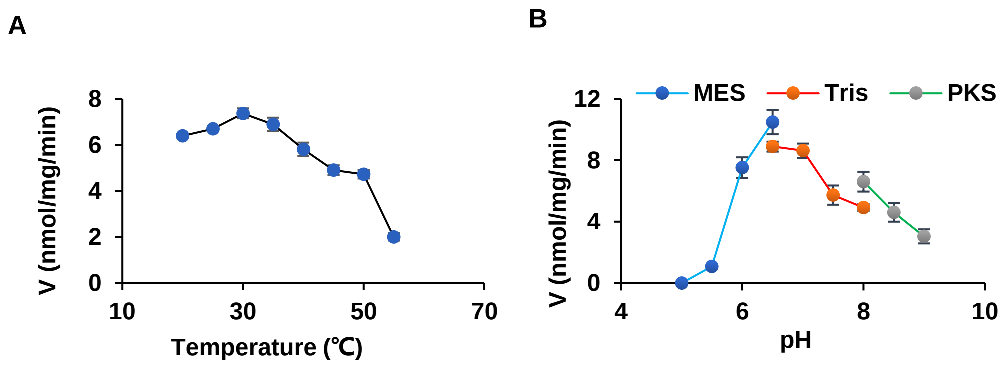

Fig. S5 Enzymatic characteristics of purified recombinant ScCGT1. Effect of various pH (A) and temperature (B) on the enzyme activities of ScCGT1 using phloretin as substrates, UDP-glucose as sugar donor.

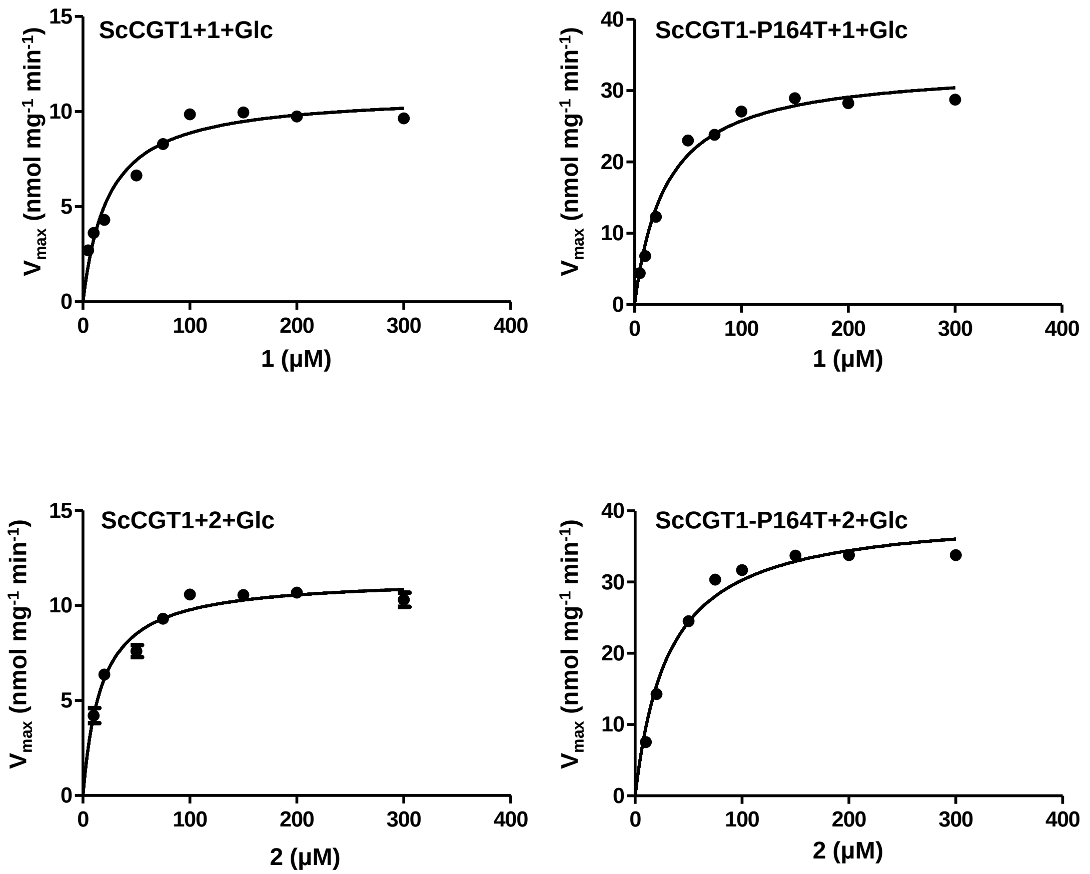

Fig. S6 Kinetic analysis of ScCGT1 and ScCGT1-P164T mutant. (**1,** phloretin; **2**, 2-hydroxynaringenin; Glc, UDP-glucose).

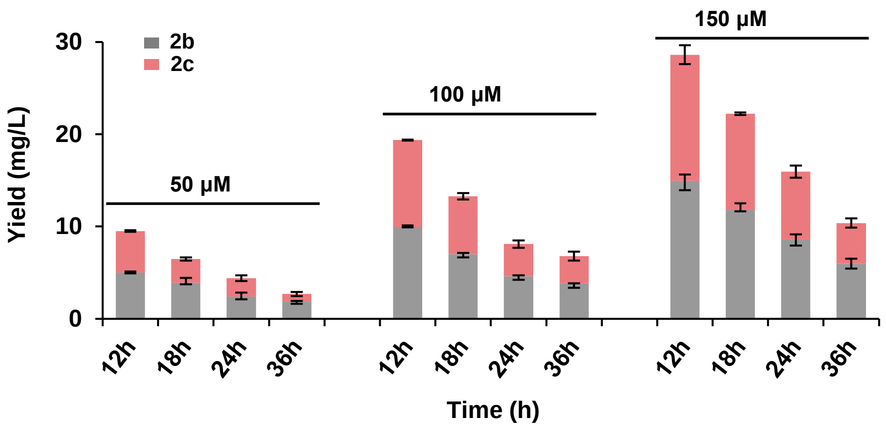

Fig. S7 The effect of varying the concentration of naringenin on the production of vitexin (**2b**) and isovitexin (**2c**).

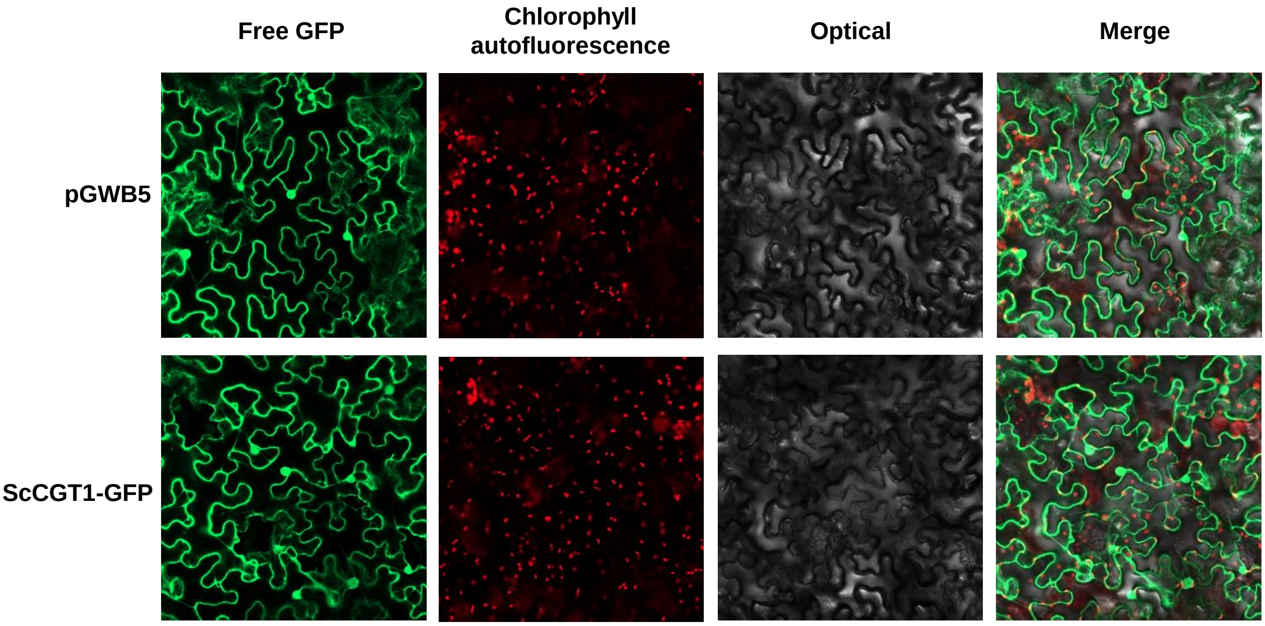

Fig. S8 Subcellular localization of *ScCGT1*. Sub-cellular localization of vector pGWB5 and the products of the transgenes ScCGT1-GFP in transiently transformed tobacco leaf discs. The GFP signal appears green and the chlorophyll signal is red.
