# Supplementary material for: Identification of a flavonoid C-glycosyltransferase from fern species Stenoloma chusanum and the application in synthesizing flavonoid C-glycosides in Escherichia coli
Source: Microb Cell Fact. 2022 Oct 14;21:210. doi: 10.1186/s12934-022-01940-z (PMC9563126; doi:10.1186/s12934-022-01940-z)
Supplement: Supplementary file 2 — Additional file 2: Table S1. The sequences of UGT genes in S. chusanum. Table S2. Accession numbers of amino acid sequences used for glycosyltransferases phylogenetic reconstruction. Table S3. The conversion rate generated by the phloretin or naringenin to produce the corresponding C-glycosides at the 100 μM substrate concentration. Table S4. The kinetic parameters of recombinant ScCGT1 and other CGTs using phloretin (1) or 2-hydroxynaringenin (2) as substrate and UDP-glucose as the sugar donor. Table S5. The primers were used for qRT-PCR analysis. Table S6. The primers were used to obtain the full length of ScGTs genes. Table S7. Sequences of specific primers for PCR. Table S8. The primers were used for the site-directed mutagenesis vector construction. Table S9. The primers used for plasmid constructions (into pETDuet-1 vector). Table S10. Primers for sub-cellular localization analysis. [file 12934_2022_1940_MOESM2_ESM.doc]

**Table S1** The sequences of UGT genes in *S. chusanum*.

| Gene | Accession No. | Nucleotide sequence |
| --- | --- | --- |
| ScGT1 | ON936072 | ATGGCGTCCTGCGACACTGGTCTCCTAAGCGACCAGCGCCACCATGCCCTCATGTTTCCCTACCCTGTGCAGGGCCACATTCAGCCTTTCATGCGCCTCTCCAAGCTCCTAGCCACCAACTATGGCTTCACCATCTCCTTCTTCAACTCTGAGGAGAACCATCGCCGGCTCCTCGCTGATCGAGATGAGCCTGCTTCTCAAGAAGACCGTGCCGAAGATGCCGAAGGCAAACCTCTCGATTTCCGCATGCTCTTTCTGCCTGACGTGCTGCCGTCCTCCACCGAAGTTGAGCAAATGTCAGCAGGCACACATATTGAGAAGAGCCATGTCTTGCTCAGCAAGACCTTTCTTAACACAGATCCGGCCGCGCTGGAAACCCTAATCAGAAGCCAGAGCCCACCCATCACATGCTTGATCCTCGACCCTTTAGTAACTGTCATTGCTCCTGGCCTTGGTGAAAAACTTGGCATCACATCCCTTATCTTCTTCCCTGCATCTGCTGCGAGCTTAGCCACGGCAATTCGAAGCGGTGAGGTCGACGGTGATGCCAAGCAGATGACTCGCATTGTTGGGGCCCCAGTCTTGGATGTGAAAGAGGTCAATCCGTTTCTCAGCATTTGCGGGCGAGGTCATTCCGACTTCATGGCCAGGTTATTCACTCAGCCTTTCAGGAAGAAACTTATCAAGGCAAATGACATCATTTTGATAAACACAGTTGAGGAACTGGAGGAAAGCACGTTGAAGCATTTGCAGGAAGAGAACAAAGTGTGTGCAATAGGACCAGTGTTACCGCCATGGGGAGCATTTGCGGTGCCGACATTGTGGTCACAGGACTACTCCTGCTTGGATTGGCTAGACAAGCAGCCTGCTGCGTCTGTTTTGTTTGTGTCCTTTGGGAGTTTAGCCACCTTGTCTTCTACCCAATTATCAGACCTGGCTCTGGGATTAGAAGCTAGCAATCAAAGGATTTTGTGGGTTACCCGGCCGGACCAAATCTATGGCAAGGCGCCGGATTTGCCATCAGATTTCCTGGAGCGCATCAAAGATAGAATACTTGTATTCTCATGGGTTCCCCAACTTCATGTTTTATGTCATACATCTGTAGGTGGTTTCCTATCACATTGTGGTTGGAACTCCACCATCGAAAGCATTGCGGCTGGTGTCCCAATCCTTGCTTGGCCTTTCTTTGGGGACCAAATGCTCAATGCCAAGTGCGTGGTGGAGAAGTGGCGCATCGGGTTGGCCTTAACCGGAGCTGGTGGTGCCATGTCAAAGGCTGTTGTGGAGACCAGGGTCAAGGATCTGATGGAGGGGGATCTCAGCAAAGAGTTGAGAAACCGTGCCCAAAACCTGAAGCATATTGTTGTCAAAGCTCTCTCTCATGGGGGCTCCTCCTCTTCAAACTTGCAAAAACTTTTTGGCCATCTCTGA |
| ScGT2 | ON936073 | ATGGTGCATGTTCTGGCGGTGCCCTTTCAAGCGGAGGGCCATGTGAATGGCTTGTTGAGGCTCGCCCGGGCGCTGGCAGCCATGGGCGTGTCGGTCACCTTCGTCTACCCGGCCCGCTTCTACGCTTTGGCAAGCAAACGTTCACAGCTCGATCGTGATGGCGATGCGTTACTGCAATTTGAGGCAGTAGAGGACGGCCTGCCCGTGGAAGAAGAGCATGTCCTCACAGCTCCGCTGTTGCATGCTTCCATCCCCTTCTTCATGAAATCTGTGAAGCACCTTTTGGATCGTCTTCTCGCCGAAGAAGATAGCTCCACATCATCTCATCTTCCTCCGCTCTCTTGCGTCATCTCTGACTGTGTGGTTCCATGGACAAAGGAGCTTGCCGATGCAGCTGGCCTGCCTCGGATCCCCTTCTGGACTTCCAGCGCGGCCTCCTATGCCATGGGTGCCCATCTTCCTCTGCTCATTTCCAGAGGTTGGGTTCCTGTACAGAATAGCTTATTGGTGCAAGGCAAGATGTGGAAGGGGGATGCTTCTCTGGTTGACTGCATACCTGGCCTACCTCCCTTTCCTATCACGGATCTACCATCCCAATTTGTGGAGGCAGCGGACCTCTCCAATCCCAGCCTACAGTTTCTTGCAGCAGCTTATGAGCATGCAAGGGAGGCCCCTTCTATCTTGATCCACTCCGTGTATGAGCTTGAATCTCAAGTCTTTGATGCTTTAAAGGCTCATGGCTTTGCCGTCTGCCCTGTAGGCCCTCTGTTCTTCCCTCCAAATTCTATGAGAAGTCATCGACACGCACAAGAAACTCTTCAATGGCTTGACAAAAAGCCCACCTCCTCCGTCGTCTATGTCGCCTTGGGCACAGCAACCCAACTCAGCCCAGCAGGACTTCTTTCTTTGGCCCTTGGTCTTGAAGCATCTGGACACCCTTTTTTGTGGGTCATCCGCACTGACAACATGCATGGTTCTCTCTCCAACACCCTTCCGGAAGGCTTCTTGGACCGCACCAGTGCTAGGGGCCTCATTGTGCCGTGGGTACCACAGGCAGAAGTGCTGCGACATGGCTCTGTGGGCGCTTTCTTCTCACACTGTGGCTGGAATTCGACCTTAGAGAGCATGTTTGAGGGAGTTCCTATGGTGGTATGCCCACAGGCAATGGAACAGAGATCCAATGCAAGGTGGATTGTGGAGCATTGGAGAATGGGAGTAGAGCTGGAGAGAGAAGTGGATGGCAGCTTCAGCAAGGATGCAGTGGAAAGGGCCTTGCATGAGGTAATGCACAAAACTTACAAGAAGGGGGCAGTTCAGGTGAAGGAGGTGACCAGGCGTGCAGTACAAGAGGAAGGAACGTCTGGTTCCAACTTGATCTGCTTAAAGCAGCAGCTGACCAAGTTCCATGCTCGTAAAAATCTGGAAAACGTGAGAGAGACAAGCTGA |
| ScGT3 | ON936074 | ATGGTCAAATATGATCTTGTGAAGCAGTGCCAAGCACTCATTGATTTGGAGCCCCTCCTTGTGAAGGTAGTGGAGGACCAACAATGCCCACCCGTGACTTGCATCATTGCCGATGTATTTGTCATATGCACACCTGCTGTGGCAAAACGGTTTCACATCCCACATCTTGTATTTTGGACACAGAGCGCAGCTTCCTACGCCACTCATACCATTGTTACACAGAATTATGCCTCCTTAAGAGGCCAGCTATTTCCTGAGGAGCTGCAATATGGAGAGGCCAAAGAAGGGAAGTTAATAAGGAACATACCAGGATGCCCACCGTTGAAGCTGAGGGAGGTGCCTACCTTCTTGCAGGCCATGGATCCTGCAGACTTTCTCTTCAATTACCTCATCCGCAATTTTGATGATGCCCTAGATGCGGACTATGTGTTGATGAACACCTTTGAGGAGCTAGAGGCAGAGAGCATGGCGTCCATTGCCAAGCCGCCCGCGCTAGCAATAGGCCCGATGCTGCCTGCGCAGTTTGTACTCAGCAGAGATGGAGATGAAGACAGAAGCAGAGATGTAATGGTGGTCGATAAAAACCCTAATTTGATTCAGAGGAGAAATGGAGTGGTTGTGGATGAAAACCCTAATTTGATGAAGAGGGGGTCCTCTCTGTGGGTTGAGGATGAGCAAGGTTGCCTGCCTTGGCTGGACCTCCAAAAACCCAAGTCCGTGCTGTATGTTTCTTTTGGTAGCATCACTTACATGTCGGCGCGTCAGATCCACGAGTTTGCACTGGGCCTTGAAGCCGGTCGATGGCCGTTTCTCATGGTCCTGCGCCCGGAGCTGACCGTCGACGACGGTGGCGGACGACGGGCGACAACCACCTTTCTCTCCGATTTCGTGCACAGAATGAGGTCACATGCAAGGGCCGCCTTTGTGGAATGGGCACCACAACTTCAAGTCCTTGCACACCCTGCAATAGGAGGCTTTCTCACCCACTGTGGCTGGAACTCCACTCTTGAGAGCATCTCCATGGGGGTGCCCATGCTTTGCTGGCCCTACTTTGCCGACCAAATGATAAACTGTAGGTGCATAGTGGATGTTTGGAAATTGGGACTTGAATTTGATACCAAAGAGCTGGAGGACGAGACTTCCAGCAATCCTATTGATAAGGATGATGATATTGATGGCACTAGCAAAAGCAAGGACAACATTACAAAAGTTGTTAGAGCCACAAGACATGAGATTGAGCGCAAGGTCGACCATCTAATGGATCCTAATTTGAGCAAAGAAATTCGAGAACGAGCTAATACATTGAGTCTAGCTGCTAAGGAAGCTTACAAGGGTCCTTGTCAACAAAATCTCAACTTGTTATTGCATTTCCTATTCACTTACCTCAAATAA |
| ScGT3 | ON936075 | ATGGCCACCACCACCTCCACCTCCACCAAGCACCAGCAGCATCATGTGGTGGCCGTGGCCGTGCCCGCGCAGGGTCATCTCACCCCGGTCATGCGCTTCTGCAACCTCCTCGCCAAGCAGGACAACCTCATCGTCACCTTCGTCGACTTCGATCCCATCCACCAGAGGCTTGAAGAGCTCCACAACCTCCACCCGCCCCCGGCCGCCGCCGCCGCCTGCGAATCTCAACCGTCCATCCGCCGCGTCCACATACCGATCCACGGCCTCGATATATCCAAGAACTTCAGCGTCTCCTTCCAGGCCTTCTTTGAAGCTCTGCGCTCCATAGCCCCCTATCTTGAGCAGCTCATCCTGGACCTCAACAGGGATGGCCCTACCGTCACCTGCCTCCTCTCCGATTTCTTCATAACTTTGCCCACCCAACAAGTCGCCGACAAATTGGGCATACCGCGCATTGTGCAGTACCCCTGCTGCGCTTCCAGGCTCCTTCTCATGCATTACCTCATGGAAGAAGTGCACTTCTCTATCCAAGAAGTGATAACGGCGGTAGCAACCACGGGGCTCGAAAGTGAAGAGGTGTTTTGCGAGGGACTTCCCGGTTTGCCAACATTATTCAACAAGGATATACCCCATTTCAAGCATGTGACAGATGATACTCTCTTTTGTTGGAAGCTTGCCACACAAGGATGGCACCTTTGTAATACTCGAGCACATGCCGTGGTAGTCAATAGCTTCGAAGAACTTGAGGCTTCCACTTTTGCAACCCTTTCACAATGCTGTAATGTGCCAATCTATGATGTAGGATTATGGACAGAGCCCCTATCCGAGGAAATGGCCACTAATTTGTGGAAAGAAGACAAAACATGCATGACGTGGCTGGACCGACAACCCGTCTCCTCGGTTCTATACATCTCCTTTGGGAGCATAACCCTCATGTCAGAAGCCCAATATAGTGAGCTCGTAGACGGTCTCCTCCTCAGCGAACAACGTTTTCTATGGGTTTTCCGGCCGGGGCTTGTTGAGAGTATTGACTACTCTGCTAGTGTGGCAGAAATCATGTCAAGATCTGAAGGTAGAGGCTTGATCATCAACTGGGCCCCACAGCTTCAAGTGCTCGAGCACCGATCGGTGGGCGGATTTCTAACACATTGTGGTTGGAACTCCACTAGCGAGTCCATTGCTCATGGTGTACCCATGCTTTGTTGGCCATACTTTGCTGATCAACCTCTGAATGCACGCCTTATTGTCGACGTCTGGCAGGTGGGACTTTCAATAATGCAGGGTTGTAGCAGGGGGAAGCACTCATTGATTGAGAGAGGTGAAATTGAGAGAGTGATTCGAGCTCTAATGGAGGGTGAAGAGGGGAACGTTGTCAGGGACAATGCCAAGAAACTGATGGGAAAAAGCTCACAATGTCTCCAAAAGAATGGCTCCTCCCACATAAAATTGAAGGCCCTGGTTGCATCTCTCTTTTAA |

**Table S2** The conversion rate generated by the phloretin or naringenin to produce the corresponding *C*-glycosides at the 100 μM substrate concentration.

| Strains | Substrates | Metabolites | Maximum conversion rate (%) |
| --- | --- | --- | --- |
| W1 | Phloretin (**1**) | Nothofagin (**1a**) | 41 |
| W2 | Phloretin (**1**) | Nothofagin (**1a**) | 87 |
| EA1 | Phloretin (**1**) | Nothofagin (**1a**) | 93 |
| EA2 | Phloretin (**1**) | Nothofagin (**1a**) | 99 |
| EA3 | Naringenin | Vitexin (**2b**) and isovitexin (**2c**) | 44 |
| EA4 | Naringenin | Vitexin (**2b**) and isovitexin (**2c**) | 62 |
| EA5 | Naringenin | Vitexin (**2b**) and isovitexin (**2c**) | 91 |

**Table S3** The kinetic parameters of recombinant ScCGT1 and other CGTs using phloretin (**1**) or 2-hydroxynaringenin (**2**) as substrate and UDP-glucose as the sugar donor.

| Enzyme | Organism | Uni-Prot or GenBank ID | Substrate | *K_m_* (μM) | *V_max_* (nmol mg^-1^ min^-1^) | *k_cat_* (s^-1^) | *k_cat_*/*K_m_*(M^-1^s^-1^) | Source |
| --- | --- | --- | --- | --- | --- | --- | --- | --- |
| ScCGT1 | *S. chusanum* | ON936072 | Phl | 23.8±2.8 | 11.0±0.3 | 0.01±0.00 | 5.6×10^2^ | This study |
| ScCGT1  -P164T | *S. chusanum* |  | Phl | 29.8±2.6 | 33.4±0.8 | 0.04±0.00 | 1.4×10^3^ | This study |
| ScCGT1 | *S. chusanum* | ON936072 | 2-OHNA | 17.5±2.0 | 11.5±0.3 | 0.01±0.00 | 8.0×10^2^ | This study |
| ScCGT1  -P164T | *S. chusanum* |  | 2-OHNA | 31.7±2.9 | 39.9±0.9 | 0.05±0.00 | 1.5×10^3^ | This study |
| SbCGTa | *Scutellaria*  *baicalensis* | MK894443 | 2-OHNA | 2.6±0.4 | 4150.0±199.0 | 3.9±0.19 | 1.5×10^6^ | [37] |
| SobCGT1 | *Sorghum*  *bicolor* | C5Z8Y8 | 2-OHNA | 28.0±7.6 | NR | NR | NR | [6] |
| SobCGT2 | *Sorghum*  *bicolor* | C5Z8Y5 | Phl | 27.1±11.6 | NR | NR | NR | [6] |

Note: Phl, phloretin; 2-OHNA, 2-hydroxynaringenin; NR, not reported

**Table S4 The primers were used for qRT-PCR analysis.**

| Primer name | Primer sequences (5' to 3') |
| --- | --- |
| ACTIN-F | CCAAGGCGAATCGTGAGAAG |
| ACTIN-R | GTTGTGAAGGAGTAGCCACG |
| ScGT1-RT-F | GCCAGGTTATTCACTCAGCC |
| ScGT1-RT-R | GACAAGGTGGCTAAACTCCC |
| ScGT2-RT-F | GCAGAGATGTAATGGTGGTC |
| ScGT2-RT-R  ScGT3-RT-F  ScGT3-RT-R  ScGT4-RT-F  ScGT4-RT-R | ATCGGAGAGAAAGGTGGTTG  GAAGAGGTGTTTTGCGAGGG  GGGGCTCTGTCCATAATCCT  GGATGCTTCTCTGGTTGACT  TTTGGAGGGAAGAACAGAGG |

**Table S5 The primers were used to obtain the full length of ScGTs genes.**

| Primer name | Primer sequences (5' to 3') |
| --- | --- |
| ScGT1-F | GTGCCTGCGAGTATCACTGT |
| ScGT1-R | GGCATAGATGCTCAGATGCT |
| ScGT2-F | ATGACAGAGGCACGGCAACC |
| ScGT2-R  ScGT3-F  ScGT3-R  ScGT4-F  ScGT4-R | ACAAGCATGTATTGGCTAAA  TAGAGAGGGAGAGTAGACGC  GCTCTTCCTGCTAGTTGTAC  TCTGGAGTCTGGAGGGAAAG  GAATAGTTTGGGGTAGTGGC |

**Table S6 Sequences of specific primers for PCR.**

| Primer name | Primer sequences (5' to 3') |
| --- | --- |
| ScGT1-BamHI-F | CGGGATCCATGGCGTCCTGCGACACTG |
| ScGT1-HindIII-R | CCCAAGCTTTCAGAGATGGCCAAAAAGT |
| ScGT2-EcoRI-F | CGGAATTCATGGTCAAATATGATCTTGT |
| ScGT2-XhoI-R | CCGCTCGAGTTATTTGAGGTAAGTGAATA |
| ScGT3-BamHI-F  ScGT3-EcoRI-R  ScGT4-SacI-F | CGGGATCCATGGCCACCACCACCTCCAC  GGAATTCTTAAAAGAGAGATGCAACCA  CGAGCTCATGGTGCATGTTCTGGCGGT |
| ScGT4-HindIII-R | CCCAAGCTTTCAGCTTGTCTCTCTCACGT |

**Table S7 The primers were used for the site-directed mutagenesis vector construction.**

| primer name | Primer sequence (5' to 3') |  |
| --- | --- | --- |
| ScCGT1-H26A-F | AGGGCGCCATTCAGCCTTTC |  |
| ScCGT1-H26A-R | GAAAGGCTGAATGGCGCCCT |  |
| ScCGT1-L143T-F | TCGACCCTACAGTAACTGTC |  |
| ScCGT1-L143T-R | GACAGTTACTGTAGGGTCGA |  |
| ScCGT1-P164T-F | ATCTTCTTCACCGCATCTGC |  |
| ScCGT1-P164T-R | GCAGATGCGGTGAAGAAGAT |  |
| ScCGT1-L301G-F | CTTTGGGAGTGGCGCCACCT |  |
| ScCGT1-L301G-R | AGGTGGCGCCACTCCCAAAG |  |
| ScCGT1-D141I/P142D-F | CTTGATCCTCATAGATTTAG |  |
| ScCGT1-D141I/P142D-R | CTAAATCTATGAGGATCAAG |  |
| ScCGT1-4M-F | GACCCTTTCTTCCTCAGCAAGAC |  |
| ScCGT1-4M-R | GAAGAAAGGGTCCTTCTCAATA |  |

**Table S8** The primers used for plasmid constructions (into pETDuet-1 vector).

| primer name | Primer sequence (5' to 3') |
| --- | --- |
| ScCGT1-D-BglII-F | GAAGATCTATGGCGTCCTGCGACACTGG |
| ScCGT1-D-KpnI-R | GGGGTACCTCAGAGATGGCCAAAAAGT |
| ScCGT1-P164T-D-BglII-F | GAAGATCTATGGCGTCCTGCGACACTGG |
| ScCGT1-P164T-D-KpnI-R | GGGGTACCTCAGAGATGGCCAAAAAGT |
| CjFNS I/F2H-BamHI-F | CGGGATCCATGGCTCCACCCGGTGTTAC |
| CjFNS I/F2H-HindIII-R | CCCAAGCTTCTATTCGGTGGCTCCTTCA |

**Table S9 Primers for sub-cellular localization analysis.**

| Primer name | Primer sequences (5' to 3') |
| --- | --- |
| ScCGT1-GFP-F  ScCGT1-GFP-R | GGGGACAAGTTTGTACAAAAAAGCAGGCTTAACCATGGCGTCCTGCGACACTGG  GGGGACCACTTTGTACAAGAAAGCTGGGTCGAGATGGCCAAAAAGTTTTTG |

**Table S10 Accession numbers of amino acid sequences used for glycosyltransferases phylogenetic reconstruction.**

| Sequence | Accession No. | Source |
| --- | --- | --- |
| AtF3Rht | AAM65321 | *Arabidopsis thaliana* |
| AtF3RT | AAM91139 | *Arabidopsis thaliana* |
| VvGT1 | AAB81682 | *Vitis vinifera* |
| PhF3GT | BAA89008 | *Petunia hybrida* |
| PfF3GT | BAA19659 | *Perilla frutescens* |
| HvF3GT | CAA33729 | *Hordeum vulgare* |
| PhCGT1 | MK616588 | *Phyllostachys heterocycla* |
| PgCGT1 | MK616592 | *Phyllostachys glauca* |
| PpCGT1  OsCGT  ZmCGT  DcaCGT  AbCGT  GgCGT  UGT708D1  MiCGT  CuCGT  FcCGT  FeCGTa  SbCGTa  SbCGTb  TcCGT1  SsClGT  NtGT2  PhA5GT  ThA5GT  PfA5GT  GhA5GT  AtF5GT  IhA5GT  AtUGT74F1  UGT89C1  AmUGT36  PfUGT2  GelF7GT  AtF7GT  FaGT7  PfUGT31  SbF7GT  AmUGT21  UGT73A10 | MK616593  FM179712  NP_001132650  QOD39011  MN747045  QGL05036  LC003312  KT200208  LC131334  LC131333  AB909375  MK894443  MK894444  MK644229  AAK54465  BAB88935  BAA89009  BAC54093  BAA36421  BAA36423  AAM91686  Q767C8  NP973682  AAP31923  BAG16513  BAG31951  BAC78438  AAL90934  Q2V6J9  BAG31952  BAA83484  BAG31950  BAG80536 | *Phyllostachys* *prominens*  *Oryza sativa*  *Zea mays*  *Dendrobium catenatum*  *Aloe barbadensis*  *Glycyrrhiza glabra*  *Glycine max*  *Glycyrrhiza glabra*  *Citrus unshiu*  *Fortunella crassifolia*  *Fagopyrum esculentum*  *Scutellaria baicalensis*  *Scutellaria baicalensis*  *Trollius chinensis*  *Solanum sogarandinum*  *Nicotiana tabacum*  *Petunia hybrida*  *Torenia hybrida*  *Perilla frutescens*  *Glandularia x hybrida*  *Arabidopsis thaliana*  *Iris hollandica*  *Arabidopsis thaliana*  *Arabidopsis thaliana*  *Antirrhinum majus*  *Perilla frutescens*  *Glycyrrhiza echinata*  *Arabidopsis thaliana*  *Fragaria ananassa*  *Perilla frutescens*  *Scutellaria baicalensis*  *Antirrhinum majus*  *Lycium barbarum* |
| TOGT1 | AAK28303 | *Nicotiana tabacum* |
| TOGT2 | AAK28304 | *Nicotiana tabacum* |
